# Ndfip-mediated degradation of Jak1 tunes cytokine signalling to limit expansion of CD4+ effector T cells

**DOI:** 10.1038/ncomms11226

**Published:** 2016-04-18

**Authors:** Claire E. O'Leary, Christopher R. Riling, Lynn A. Spruce, Hua Ding, Suresh Kumar, Guoping Deng, Yuhong Liu, Steven H. Seeholzer, Paula M. Oliver

**Affiliations:** 1 Perelman School of Medicine, University of Pennsylvania, Philadelphia, Pennsylvania 19104, USA; 2 Department of Pathology and Laboratory Medicine, Cell Pathology Division, The Children's Hospital of Philadelphia, Philadelphia, Pennsylvania 19104, USA; 3 Progenra Inc, Malvern, Pennsylvania, 19355, USA; 4 Department of Pediatrics, The Children's Hospital of Philadelphia, Philadelphia, Pennsylvania 19104, USA

## Abstract

Nedd4 family E3 ubiquitin ligases have been shown to restrict T-cell function and impact T-cell differentiation. We show here that Ndfip1 and Ndfip2, activators of Nedd4 family ligases, together limit accumulation and function of effector CD4+ T cells. Using a three-part proteomics approach in primary T cells, we identify stabilization of Jak1 in Ndfip1/2-deficient T cells stimulated through the TCR. Jak1 degradation is aborted in activated T cells that lack Ndfips. In wild-type cells, Jak1 degradation lessens CD4+ cell sensitivity to cytokines during TCR stimulation, while in Ndfip-deficient cells cytokine responsiveness persists, promoting increased expansion and survival of pathogenic effector T cells. Thus, Ndfip1/Ndfip2 regulate the cross talk between the T-cell receptor and cytokine signalling pathways to limit inappropriate T-cell responses.

## Abstract (web summary)

Ndfip1 is an activator of Itch E3 ubiquitin ligase that limits T cell activation. Here the authors identify Jak1 in a proteomic screen for Ndfip dependent substrates, and show that Ndfip1/2 double-deficient T cells have reduced degradation of Jak1 and as a result are hyper-responsive to cytokine stimulation.

## Graphical abstract

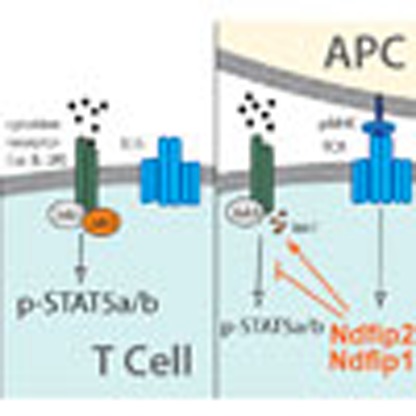

Integration of signals from T-cell receptor (TCR), co-receptors and cytokine receptors directs proliferation, survival and differentiation of T cells. Cross talk among these pathways is essential to prevent aberrant T-cell responses. One example of such cross talk is TCR-induced downregulation of cytokine receptor signalling to limit cytokine responses^1^,^2^,^3^,^4^. Ubiquitylation of protein substrates by E3 ubiquitin ligases can regulate both TCR and cytokine receptor signalling. Several members of the Nedd4 family of E3 ligases have known roles in T cells, including limiting T_H_2 differentiation, regulating activation, and promoting anergy^5^,^6^,^7^,^8^,^9^. However, as unbiased screens for identification of E3 ligase substrates, particularly in primary cells, are rare, only a handful of protein targets for Nedd4 E3 ligases have been identified using targeted approaches. To date, published substrates of these E3 ligases include TCR signalling intermediates and TCR-activated transcription factors^5^,^6^,^7^,^8^,^9^.

In mice, loss of function of the Nedd4 family member Itch results in CD4+ T-cell hyperactivation and T_H_2 cytokine production, leading to spontaneous inflammation^5^,^10^. Similar immunopathology is observed in humans with a loss of function mutation in Itch^11^. *In vitro*, catalytic activity of Itch and related ligases is potentiated by, or requires interaction with, Nedd4 family interacting protein 1 (Ndfip1) and Ndfip2 (ref. 12). Loss of function mutations in mice support that Ndfip1 activates Itch *in vivo* to limit T cell activation and T_H_2 differentiation^13^,^14^,^15^. *In vitro* binding and ubiquitylation assays suggest that Ndfip1 and Ndfip2 are both sufficient to activate the catalytic function of Nedd4-family E3 ligases^12^,^16^,^17^,^18^,^19^; however, an *in vivo* role for Ndfip2 is unknown.

Here we establish a role for Ndfip2 in regulating immune responses. Although *Ndfip2*−/− mice show no overt immunopathology, animals with T cells lacking both Ndfip1 and Ndfip2 have a dramatically increased pool of activated CD4+ T cells compared with mice with T cells lacking only Ndfip1. To determine the mechanism whereby loss of Ndfip1/Ndfip2 drives expansion of effector cells, we developed an unbiased and reproducible proteomic workflow for use in primary T cells. Consistent with published data, this proteomic approach confirmed that the E3 ligases Itch and Nedd4-2 (also called Nedd4L) are active in an Ndfip-dependent manner in stimulated effector T cells^8^,^12^,^13^,^19^. We identified several candidate substrates for Ndfip-dependent degradation, and validated that Jak1 is aberrantly degraded in T cells lacking Ndfip1/Ndfip2. Jak1 is ubiquitylated on multiple lysine residues and degraded following TCR stimulation of WT cells; this degradation is aborted in Ndfip-deficient cells. Consistent with increased stability of Jak1, T cells lacking Ndfip1/Ndfip2 fail to undergo TCR-mediated downregulation of cytokine responsiveness, a Jak1-dependent process, as measured by STAT5 phosphorylation following exposure to IL-2. Increased Jak1 signalling led to increased survival and proliferation of these cells *in vitro*, while *in vivo* this drives an expanded population of pathogenic effector T cells.

Our data reveal that TCR-induced cytokine non-responsiveness requires Ndfip-dependent degradation of Jak1. This is a previously unknown function for Ndfips in restricting cytokine signalling to limit expansion, and, consequently, pathogenicity, of CD4+ effector T cells.

## Results

### Generation of Ndfip2 knockout/GFP knock-in mice

Given that deficiency in either Itch or Ndfip1 leads to hyperactive T cells and T_H_2-mediated pathology^5^,^13^,^15^, and knowing that Ndfip1 and Ndfip2 have similar functions *in vitro*^12^,^16^,^17^,^18^,^19^, we investigated whether Ndfip2 might also play a role in T cells. We generated *Ndfip2* knockout mice by insertion of GFP into exon 2 of the *Ndfip2* gene, putting subsequent exons out of frame (Supplementary Fig. 1a–c). We observed Mendelian frequencies of *Ndfip2*−/− mice (Supplementary Fig. 1d), in contrast to the sub-Mendelian frequency of *Ndfip1*−/− mice (Supplementary Fig. 1e), at weaning.

Using *Ndfip2*+/− mice, which express one copy of GFP under the *Ndfip2* promoter, we analysed GFP as a reporter of Ndfip2 expression. In splenocytes, we observed the highest GFP expression in T cells (Supplementary Fig. 2a). In stimulated *Ndfip2*+/− CD4+ T cells, GFP^high^ cells increased in abundance over the course of stimulation and corresponded with CD44 expression, high CD25 expression and cell division (Supplementary Fig. 2b,c).

### *Ndfip2*−/− mice do not show signs of inflammation

**Figure 1:**
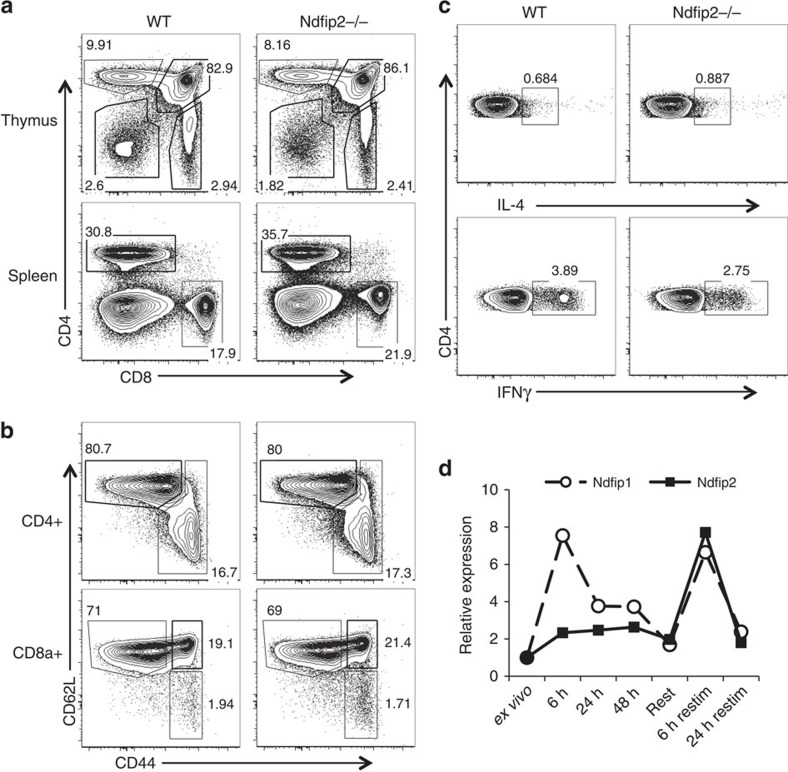
*Ndfip2*−/− mice do not show signs of inflammation. (**a**,**b**) Representative flow cytometry analysis of T-cell populations from thymus and spleen of 5–7-week-old *Ndfip2*−/− and age-matched control mice: (**a**) CD4+ and CD8+ cells, (**b**) CD44 and CD62L expression on these cells, as noted. (**c**) Intracellular cytokine staining for IL-4 and IFNγ in CD4+ T cells from *Ndfip2*−/− and WT spleens stimulated *ex vivo* with PMA and ionomycin in the presence of BFA. Representative of at least five mice per genotype, 5–7 weeks of age. (**d**) CD4+ T cells were stimulated *in vitro* for the indicated time periods with αCD3/CD28. *Ndfip1* and *Ndfip2* expression was analysed by qPCR. *Ndfip1/Ndfip2* expression relative to *Actb* was normalized to expression in unstimulated CD4+ T cells. Representative of a minimum of three independent experiments.

As our GFP reporter indicated high Ndfip2 expression in T cells, we focused our analysis of *Ndfip2*−/− mice on the T-cell compartment. *Ndfip2*−/− mice had normal thymic populations and normal CD4+ and CD8+ T cell percentages in lymph nodes and spleens (Fig. 1a; Supplementary Fig. 3a). *Ndfip2*−/− mice had no increase in percent of CD44+ T cells or cytokine production upon *ex vivo* analysis (Fig. 1b,c; Supplementary Fig. 3a). Helper T-cell differentiation *in vitro*, measured by expression of lineage defining transcription factors and cytokine production, was similar between *Ndifp2*−/− and control cells (Supplementary Fig. 3b,c).

If Ndfip1 and Ndfip2 have overlapping molecular functions, expression of Ndfip1 might mask effects of Ndfip2 in immune cells. *Ndfip1* mRNA expression is increased on T-cell activation, consistent with its role limiting aberrant activation and cytokine production in stimulated naive CD4+ T cells^13^,^20^. Comparing expression of *Ndfip1* and *Ndfip2* mRNA during CD4+ T cell stimulation revealed that Ndfip1 was more robustly induced on initial stimulation than Ndfip2 (Fig. 1d). However, *Ndfip1* and *Ndfip2* both increased in expression following re-stimulation. Together with our GFP reporter data, these data support that *Ndfip2* expression is increased in newly activated CD4+ T cells, but more strongly induced during stimulation of previously activated T cells.

### Ndfip2 deficiency exacerbates inflammation in Ndfip1 cKO mice

To test whether Ndfip1 expression in *Ndfip2*−/− mice masked effects of Ndfip2 deficiency, we generated mice doubly deficient in Ndfip1 and Ndfip2. The number of double knockout (DKO) pups was unexpectedly low at weaning, and foetal analysis indicated sub-Mendelian frequencies (Supplementary Table 1). We next generated mice doubly deficient for Ndfip1 and Ndfip2 in T cells by crossing *Ndfip2*−/− mice to *Ndfip1*^fl/+^CD4 Cre+ mice. *Ndfip*1^fl/fl^CD4 Cre+ mice (cKO) exhibit T-cell-mediated, T_H_2-biased inflammation similar to that observed in *Ndfip1*−/− mice^20^. We hypothesized that loss of Ndfip2 in Ndfip1 cKO mice would worsen the Ndfip1 cKO phenotype if Ndfip2 acts to prevent aberrant T-cell responses.

**Figure 2:**
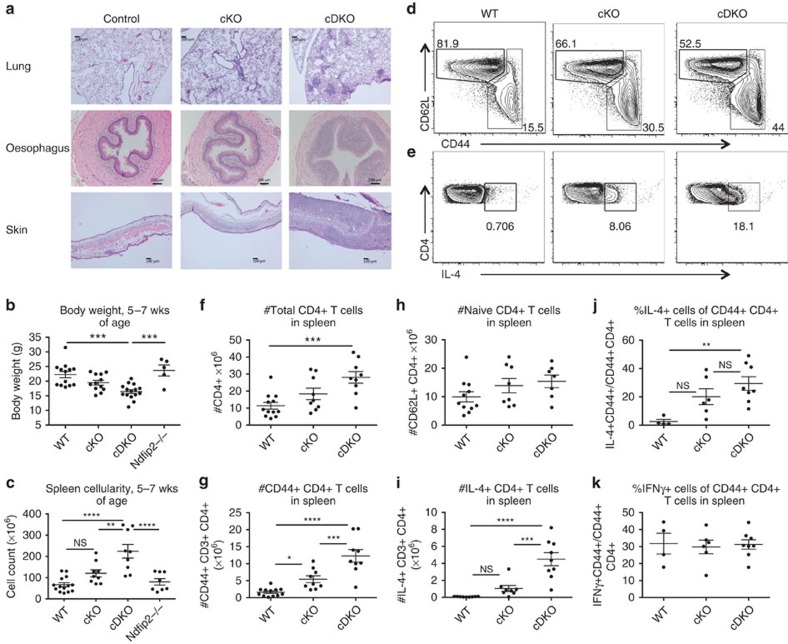
Ndfip2 deficiency exacerbates inflammation in *Ndfip1*^fl/fl^CD4Cre+ mice. (**a**) H&E-stained sections of oesophagus, lung and skin from representative 8-week-old control, *Ndfip1*^*fl/fl*^CD4 Cre+ (cKO) and *Ndfip2*−/− *Ndfip1*^*fl/fl*^CD4 Cre+ (cDKO) mice (bar represents 100 μm). (**b**,**c**) Body weight (**b**) and (**c**) spleen count of 5–7-week-old WT, cKO, *Ndfip2*−/− and cDKO mice. Mean±s.e.m. *n*=5–15 mice. (**d**,**e**) Representative flow cytometry analysis of splenic CD3+CD4+ T cells, showing (**d**) expression CD44 and CD62L and (**e**) intracellular levels of IL-4 after *ex vivo* stimulation with PMA/ionomycin in the presence of BFA. (**f**–**i**) Quantification of (**f**) the number of CD4+ T cells, (**g**) naive CD62L^high^ CD4+ T cells, (**h**) CD44+ CD4+ T cells and (**i**) IL-4+ CD4s from spleen analysed by flow cytometry. Mean±s.e.m., *n*=7–12 mice. (**j**,**k**) Quantification of percent IL-4+ or IFNγ+ CD4+ T cells among CD44 high T cells. Mean±s.e.m., *n*=4–7 mice. *P* values calculated by one-way ANOVA with Holm–Sidak test for multiple comparisons: *P<0.05, ^**^P<0.01, ^***^P<0.001, ^****^P<0.0001.

We observed that *Ndfip2*−/−*Ndfip1*^fl/fl^CD4 Cre+ (referred to as cDKO) mice showed increased inflammation at barrier surfaces, increased spleen size and decreased body weight relative to age-matched cKO, *Ndfip2*−/− and control mice (Fig. 2a–c). Splenic CD4+ T cells from cDKO mice were more likely to express CD44 (Fig. 2d) and produce IL-4 (Fig. 2e,i). We observed a significantly increased number of CD4+ T cells in spleens from cDKO mice (Fig. 2f) due largely to an increased number of CD44+ CD4+ T cells (Fig. 2g); the number of CD62L+ CD4+ T cells in cDKO spleens was not statistically different from cKO and control mice (Fig. 2h).

To separate the observed increase in IL-4 producing T cells from the expanded population of activated CD4+ T cells in cDKO mice, we examined cytokine production by CD44+ cells. cDKO CD44+ CD4+ T cells were somewhat more likely to produce IL-4 than cKO CD44+ CD4+ T cells (Fig. 2j), but this was not statistically significant. Among cDKO, cKO and control mice there was no difference in the likelihood of CD44+ CD4+ T cells to produce IFNγ (Fig. 2k). Together, these data indicate that loss of Ndfip2 in mice with Ndfip1-deficient T cells drives expansion of effector CD4+ T cells with pathogenic T_H_2 activity. Thus, Ndfip2, like Ndfip1, negatively regulates T-cell immune responses.

### Ndfip deficiency causes intrinsic CD4+ effector T cell expansion

Our phenotypic analysis of *Ndfip2*−/−*Ndfip1*^*fl/fl*^CD4Cre+ mice suggested that loss of both Ndfip1 and Ndfip2 in T cells could lead to expansion of pathogenic T_H_2 effector T cells, or that loss of Ndfip2 in non-T-lineage cells could drive increased expansion and pathogenicity of Ndfip1-deficient T cells. To differentiate between these possibilities, we generated mixed foetal liver chimeras. These were analysed 6 weeks after reconstitution due to inflammatory disease in DKO/WT cell recipients.

**Figure 3:**
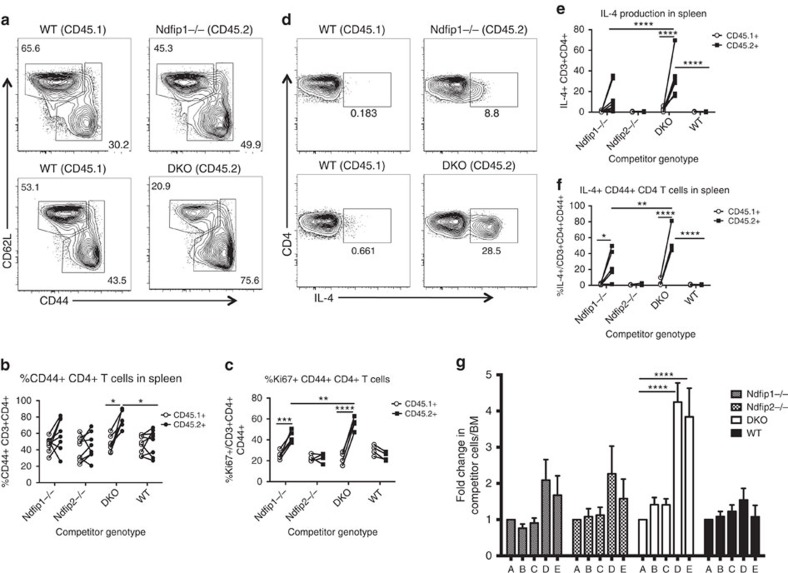
Ndfip deficiency causes intrinsic CD4+ effector T-cell expansion. (**a**–**g**) Mixed foetal liver chimeras using CD45.2 WT, *Ndfip1*−/−, *Ndfip2*−/− or *Ndfip1/Ndfip2* DKO foetal liver were analysed 6 weeks following reconstitution. (**a**) Representative CD44 and CD62L staining of splenic CD4+ T cells from Ndfip1−/− mixed chimera (top) and DKO mixed chimera (bottom), previously gated on live, singlet CD3+CD4+CD45.1 or CD45.2+ cells. (**b**,**c**) Flow cytometry analysis of CD45.1+ and CD45.2+ splenic T cells from chimeras showing (**b**) percentages of CD44+ cells and (**c**) percentages of CD44+ that are Ki67+. (**d**–**f**) *Ex vivo* stimulated splenocytes were stained for IL-4. (**d**) Representative flow plots of IL-4+ CD4+ T cells, (**e**) combined data from **d**, (**f**) percentages of CD44+ CD4 T cells that are IL-4+. (**g**) Percentages of CD45.2 cells among various T-cell subsets, normalized for reconstitution, as determined by the ratio for CD45.2:CD45.1 IgM+B220+ B cells in the bone marrow. Compartments analysed are as follows: A=IgM+B cells in bone marrow, B=double positive thymocytes, C=single positive CD4+ thymocytes, D=CD44+ CD4+ T cells in spleen, E=CD44+ CD4+ T cells in lung. Data shown in **b** and **e** were pooled from two experiments, 7–8 chimeras per group; (**c**,**f**,**g**) have 4–5 chimeras per group. Quantifications are average ±s.e.m. *P* values calculated by two-way ANOVA (**b**,**c**,**e**,**f**) or repeated measures one-way ANOVA (**g**), with Holm–Sidak test for multiple comparisons: *P<0.05, ^**^P<0.01, ^***^P<0.001, ^****^P<0.0001.

In these chimeras, DKO CD4+ T cells were more likely to be CD44+ compared with WT CD4+ T cells within the same host. The likelihood of being CD44+ was significantly higher for DKO CD4+ T cells compared with WT cells in the same host, but only trended to be higher for *Ndfip1*−/− CD4+ T cells relative to their WT counterparts (Fig. 3a,b). CD44+ DKO CD4+ T cells were significantly more proliferative, as indicated by Ki67 staining (Fig. 3c). This was also true for *Ndfip1*−/− CD4+ T cells, though to a lesser extent. As previously published, loss of Ndfip1 was sufficient to drive IL-4 production^15^; further loss of Ndfip2 resulted in an increased proportion of CD4+ T cells producing IL-4, even among CD44+ cells, relative to Ndfip1-deficient cells (Fig. 3d–f). Thus, T cells lacking both Ndfip1 and Ndfip2 are much more likely than either their WT counterparts or T cells lacking only Ndfip1 to be activated *in vivo*, and, once activated, are more proliferative and more likely to produce cytokine.

The increased percent of activated Ndfip-deficient CD4+ T cells suggested a competitive advantage of Ndfip deficiency among activated cells. We analysed this by determining the relative frequencies of CD45.2/CD45.1 cells in different T-cell populations, normalizing to the chimerism observed in IgM+ B cells from bone marrow. We found a significant increase in the relative frequency of CD44+ CD4+ DKO T cells isolated from spleen or lung (Fig. 3g). We observed a similar pattern for *Ndfip1*−/− and, surprisingly, *Ndfip2*−/− CD4+ T cells, although this did not achieve statistical significance. Thus, the population of previously activated CD4+ T-effector cells is significantly expanded when both Ndfip1 and Ndfip2 are lacking.

### Ndfip1/Ndfip2-deficient CD4+ T cells cause increased colitis

**Figure 4:**
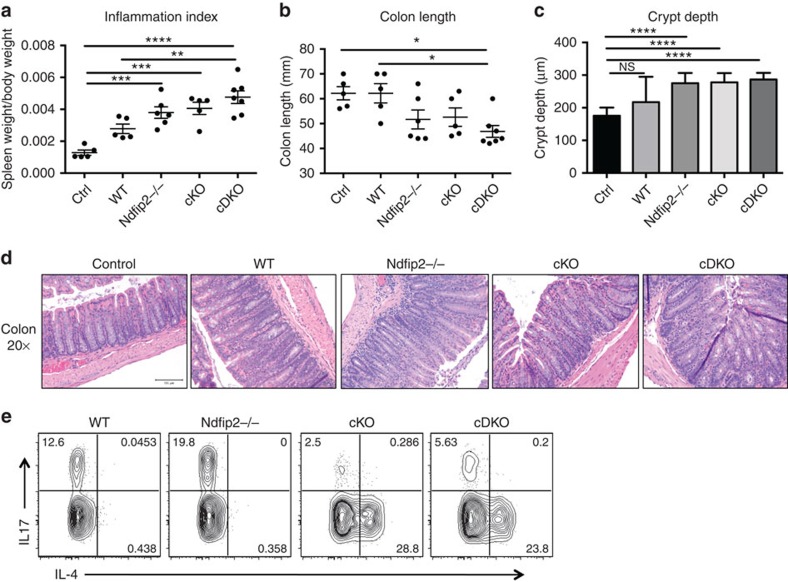
Ndfip1/Ndfip2-deficient CD4+ T cells cause increased colitis. (**a**–**e**) 0.5 × 10^6^ sorted naive CD4+ T cells from WT, *Ndfip2*−/−, cKO and cDKO mice were transferred into 6-week-old *Rag1*−/− recipients. Mice were weighed twice weekly and killed 6 weeks after transfer when 20% weight loss was observed in multiple mice. Spleen weight and body weight were compared to generate an inflammation index (**a**) and colons were measured (**b**). H&E-stained sections of the distal colon were imaged on the × 20 objective, and crypt depth was quantified (**c**,**d**). Splenocytes were stained for intracellular IL-4 and IL-17 after *ex vivo* stimulation with PMA/ionomycin in the presence of BFA, and analysed by flow cytometry (**e**). Previously gated on live singlets, CD4+, dump gate-. Quantifications shown ±s.e.m. *n*=5–7 mice. Control (ctrl) mice did not receive T cells. *P* values calculated by ordinary one-way ANOVA with Holm–Sidak test for multiple comparisons: *P<0.05, ^**^P<0.01, ^***^P<0.001, ^****^P<0.0001.

Our data indicate that both Ndfip1 and Ndfip2 control effector cell numbers and pathogenicity. To test this we transferred naive WT, *Ndfip2*−/−, Ndfip1 cKO and cDKO CD4+ T cells into *Rag1*−/− recipients to induce colitis. cDKO CD4+ T cells caused severe pathology: recipients had high spleen/body weight ratios, contracted colons and increased colon crypt depth (Fig. 4a–d). Recipients of *Ndfip2*−/− CD4+ T cells showed equivalent pathology to Ndfip1 cKO recipients—both cohorts developed more severe inflammation than WT cell recipients as measured by inflammation index, although colon histopathology, as quantified by crypt depth, was similar. *Ndfip2*−/− T cells, like WT cells, were IL-17A producers, while cDKO cells, like Ndfip1-deficient T cells, produced IL-4 (Fig. 4e). Thus, while Ndfip1 plays a dominant role in limiting T_H_2 differentiation, after initial activation both Ndfip1 and Ndfip2 limit the pathogenic potential of activated CD4+ T cells by limiting their expansion and function.

### Ndfip1/Ndfip2-deficient T cells outcompete WT cells *in vitro*

We then sought to model these phenotypic findings *in vitro*. When naive CD4+ T cells were examined after 5 days of *in vitro* stimulation, cDKO cells showed increased GATA3 expression and proliferation relative to control cells (Supplementary Fig. 4a–c). We also observed an increase in cDKO cell viability relative to experiment-matched control cells (Supplementary Fig. 4d).

We next tested whether Ndfip-deficient CD4+ T cells could outcompete WT cells within the same cytokine environment. WT cells co-cultured with cDKO CD4+ T cells had increased GATA3 expression and proliferation, but this was significantly decreased relative to cDKO cells within the same culture (Supplementary Fig. 4e–i). cDKO cells continued to show enhanced viability—on day 5 cDKO CD4+ T cells significantly out-numbered WT cells in the same culture (Supplementary Fig. 4h,i). Thus, increased cytokine production in the absence of Ndfips is insufficient to explain increased viability and proliferation.

These data suggest that loss of both Ndfip1 and Ndfip2 leads to increased survival and proliferation of activated CD4+ T cells, resulting, *in vivo*, in an expanded population of previously activated effector cells. Furthermore, Ndfip-deficient CD4+ T cells outcompete WT cells within the same cytokine milieu, suggesting that Ndfip-deficient T cells are more competent to respond to cytokines promoting division and survival.

### Identification of differential ubiquitylation by proteomics

**Figure 5:**
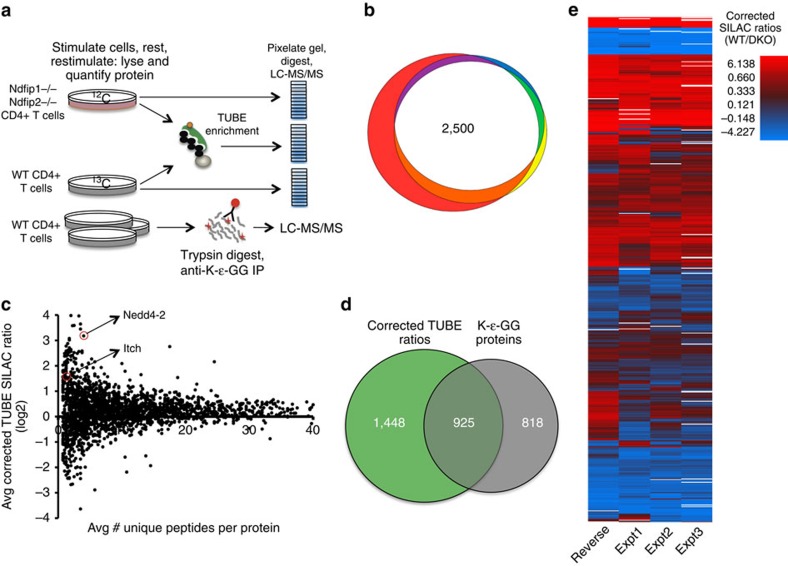
Identification of differential ubiquitylation by proteomics. (**a**) Schematic of the three proteomics methods used. (**b**) Area proportional Venn diagram illustrating the reproducibility of proteins identified as having SILAC ratios after TUBE enrichment in three out of four biological replicates. (**c**) SILAC–TUBE ratios (WT/DKO) for each protein were corrected using ‘input' ratio (DKO/WT) as calculated by label-free quantification of whole proteome DKO and WT data sets. The average corrected ratio is plotted against the average number of unique peptides observed per protein across four whole proteome LC-MS/MS experiments. Plot limited to 40 average unique peptides for clarity. Itch and Nedd4-2 (indicated) have log2 transformed corrected ratio >1 at the protein level. Itch=1.57±0.17 and Nedd4-2=3.18±0.226. (**d**) Overlap of proteins with corrected SILAC–TUBE ratios (observed in at least three experiments) and proteins identified with at least one K-ɛ-GG peptide (in at least one of the three K-ɛ-GG immunoprecipitation experiments). (**e**) Heat map illustrating reproducibility of corrected SILAC–TUBE ratios (WT/DKO, log2 transformed, observed in at least three of four experiments) for proteins identified with K-ɛ-GG peptides. Reverse=SILAC labeling swapped.

Having determined that Ndfip1 and Ndfip2 both negatively regulate activated effector CD4+ T cells, we wanted to understand the mechanism underlying the increased proliferation and survival of Ndfip-deficient CD4+ T cells. Both Ndfip1 and Ndfip2 have been shown *in vitro* to bind and activate several members of the Nedd4 family of E3 ubiquitin ligases. While putative substrates of Nedd4 family E3 ligases have been described in targeted studies, in primary lymphocytes unbiased screens to identify substrates of these and other ubiquitin ligases are lacking. To address this need, we developed a three-part proteomic workflow to identify Ndfip-dependent ubiquitylation in activated CD4+ T cells (Fig. 5a).

We first used stable isotope labelling of amino acids in cell culture (SILAC) in combination with tandem ubiquitin binding entities (TUBEs)^21^ to enrich polyubiquitylated proteins from mixed WT and Ndfip-deficient cell lysates. We reproducibly obtained SILAC ratios (WT/DKO) for ∼2,500 proteins (Fig. 5b). In parallel, we performed whole proteome analysis of DKO and WT CD4+ T cells. We then calculated the unenriched ‘input' ratio (DKO/WT) for each protein and applied this correction to the TUBE-enriched SILAC ratio for the same protein across each biological replicate (Fig. 5c; Supplementary Fig. 5a,b and Supplementary Data 1).

Peptides derived from trypsin cleavage of ubiquitylated proteins contain a di-glycine remnant (K-ɛ-GG) on modified lysine residues^22^. The vast majority of such diglycine peptides are derived from ubiquitin linkages^23^. To limit our analysis to proteins directly modified by ubiquitin, we utilized an antibody against the K-ɛ-GG motif^24^,^25^ to enrich modified peptides from activated WT CD4+ T cells (Supplementary Data 2).

Analysing proteins with at least three TUBE–SILAC ratios and at least one modified lysine (∼1,000 proteins, Fig. 5d) indicated good reproducibility across replicates (Fig. 5e; Supplementary Data 3). Thus, despite technical limitations imposed by analysing primary lymphocytes, this approach yields reproducible data with good depth of proteome coverage.

### Ndfip-dependent function of Nedd4 family ligases in T cells

Ndfip1 and Ndfip2 bind and activate the enzymatic function of several Nedd4 family E3 ubiquitin ligases *in vitro*; once active, these ligases promote their own ubiquitylation^12^,^16^,^17^,^18^,^26^. Thus, in effector T cells K-ɛ-GG peptides should be observed for active Nedd4 family ligases; if these same ligases depend on Ndfips for function, increased ubiquitylation in WT cells would result in high WT/DKO TUBE–SILAC ratios. We therefore sought to validate our proteomics screen by identifying Nedd4 family E3 ligases with Ndfip-dependent function in effector T cells.

Among Nedd4 family members, we consistently observed Itch, Nedd4-2 and WWP2 after TUBE immunoprecipitation; analysis of the whole proteome revealed increased abundance of Itch and Nedd4-2 in DKO T cells, while WWP2 was not consistently identified (Supplementary Data 1). Of the Nedd4 family ligases, only Itch and Nedd4-2 were observed following diglycine remnant immunoprecipitation (Supplementary Data 2). Correcting the TUBE–SILAC ratios (WT/DKO) for both Itch and Nedd4-2 to account for the increased abundance of these ligases in Ndfip-deficient T cells indicated significantly more ubiquitylation in WT cells compared with Ndfip-deficient cells (Fig. 5c; Supplementary Fig. 5c).

**Figure 6:**
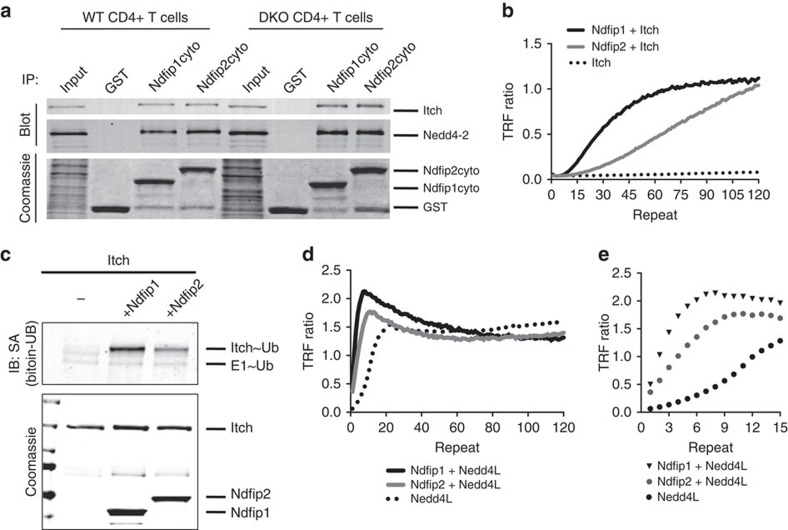
Ndfip1 and Ndfip2 promote Itch and Nedd4-2 activity. (**a**) Immunoblot of Itch and Nedd4-2 isolated from activated WT or Ndfip1/Ndfip2 DKO CD4+ T-cell lysates by GST pulldown of Ndfip1 and Ndfip2 cytosolic domain GST fusion proteins. Coomassie stain for the GST fusion proteins revealed full-length products for each construct as well as a GST cleavage product. (**b**) Itch activity, in the absence or presence of Ndfip1 or Ndfip2, was analysed via TR-FRET polyubiquitylation assay. (**c**) E2/E3 transthiolation assay was used to test whether Ndfip2 promotes ubiquitin ‘charging' of Itch. Biotinylated ubiquitin non-covalently bound to Itch was analysed by western blot using fluorescent streptavidin (top); total protein was visualized by Coomassie stain (bottom). (**d**,**e**) As in **b**, human Nedd4L activity was assessed alone or in the presence of Ndfip1 or Ndfip2 by TR-FRET. Data for each panel is representative of a minimum of three independent experiments.

Itch is known to interact with, and be activated by, Ndfip1 in T cells, while Nedd4-2 has been shown to work with either Ndfip1 or Ndfip2 (refs 8, 13). In Ndfip doubly deficient T cells, decreased activity of Itch/Nedd4-2 could indicate E3 ligase dependence on both Ndfip1 and Ndfip2, or either Ndfip1 or Ndfip2 alone. We first assessed binding of Ndfips to Nedd4 family E3 ligases using the cytoplasmic domains of either Ndfip1 or Ndfip2 to isolate the endogenous E3 ligases from T-cell lysates. Both Ndfip1 and Ndfip2 were capable of isolating Itch and Nedd4-2 from WT CD4+ T cells in this pulldown assay (Fig. 6a). Analysis of Ndfip1/2-deficient cells yielded similar results (Fig. 6a).

Next, we tested the ability of Ndfip1 and Ndfip2 to activate Itch enzymatic function, which is restrained through autoinhibition^12^,^27^,^28^. Using a time-resolved fluorescence resonance energy transfer (TR-FRET) assay^21^, we observed Itch ubiquitylation activity only in the presence of Ndfip1 or Ndfip2 (Fig. 6b). While unequal purity of the GST fusion Ndfips made quantitative comparisons of activation difficult (Supplementary Fig. 5d), we can conclude that both Ndfip1 and Ndfip2 are sufficient to activate the E3 ligase function of Itch. We recently determined that Ndfip1 activates Itch catalytic activity by promoting ubiquitin charging of Itch^29^. However, the role of Ndfip2 in this process is unknown. We found that either Ndfip1 or Ndfip2 is sufficient for E2-mediated ubiquitin charging of Itch (Fig. 6c), indicating that Ndfip1 and Ndfip2 activate Itch via the same molecular mechanism. Thus, both Ndfips could promote the ubiquitylation activity of Itch observed in our proteomic analysis.

We then assessed whether Nedd4-2 is activated by Ndfips. Under our cell-free *in vitro* conditions using the human homologue of Nedd4-2, Nedd4L, we observed that Nedd4L was not autoinhibited; nevertheless, an increased rate of Nedd4L activity was evident when Ndfip1/Ndfip2 were included in the reaction (Fig. 6d,e). Thus, while Ndfips are not required for function of Nedd4L in these assays, they potentiate its catalytic activity. Both Itch and Nedd4-2/Nedd4L have been published to work with Ndfips in T cells, and are known to autoubiquitylate when activated, indicating that our assay can identify Ndfip-dependent ligase activity and changes in Ndfip-dependent ubiquitylation in an unbiased fashion^8^,^12^,^13^,^15^,^19^,^29^.

### Jak1 degradation is dependent on Ndfip1/Ndfip2

Having validated that our screen can identify Ndfip-dependent ubiquitylation, we turned to substrate identification. Candidate substrates of Ndfip-dependent ubiquitylation (Supplementary Data 4) had positive corrected TUBE–SILAC ratios (WT/DKO) in all replicates, low error across replicates, and multiple K-ɛ-GG peptides. We identified Jak1 and Jak2 as differentially ubiquitylated in Ndfip-deficient cells, and observed K-ɛ-GG peptides from all Jak family members (Supplementary Fig. 6a,b). Analysis of Jak2, which is known to be monoubiquitylated, and for which suppressor of cytokine signalling (SOCS)-mediated degradation has been demonstrated^30^, revealed that Jak2 is not robustly degraded during TCR stimulation (Supplementary Fig. 6c,d). Although Jak1 ubiquitylation has never been shown in T cells, it is known to have a short half-life^3^. Consistent with ubiquitylation of Jak1, we observed that several distinct lysines in Jak1 had the K-ɛ-GG motif. Jak1 is a key component in signalling via common γ chain containing cytokine receptors, and can drive survival, proliferation and differentiation of activated T cells^31^,^32^,^33^,^34^,^35^,^36^. Therefore, we hypothesized that Jak1 degradation in CD4+ T cells depends on Ndfip1 and Ndfip2.

**Figure 7:**
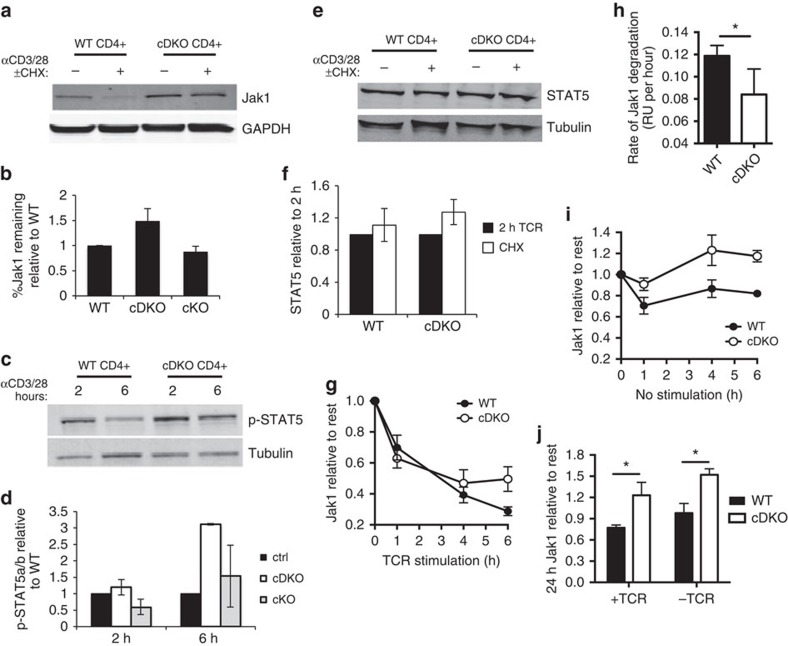
Jak1 degradation is dependent on Ndfip1/Ndfip2. (**a**–**j**) Immunoblotting of restimulated WT and cDKO CD4+ T cells. (**a**) Cycloheximide was added 2 h after CD3/CD28 stimulation; cells were then incubated for an additional 4 h. (**b**) Level of Jak1 was normalized to GAPDH. The stability of Jak1 was determined by normalizing the percent Jak1 remaining in cDKO or cKO cells to the percent remaining in experiment-matched control cells. Data shown are average ±s.e.m. from three to five biologic replicates in more than three experiments. (**c**,**d**) p-STAT5 was quantified relative to tubulin. Relative levels of p-STAT5 in cDKO and cKO CD4+ T cells were normalized to experiment-matched control cells at 2 and 6 h of restimulation. Data shown is average ±s.e.m. for two biologic replicates. (**e**,**f**) Immunoblotting of total STAT5 in restimulated WT and cDKO CD4+ T cells. Cells were stimulated for 2 h, and cycloheximide was added for an additional 2 h of stimulation. (**f**) Level of STAT5 was normalized to GAPDH. The stability of STAT5 was determined by normalizing the relative STAT5 remaining after stimulation to the amount of STAT5 at 2 h. Data shown are average ±s.e.m. from three biologic replicates. (**g**–**j**) Levels of Jak1, normalized to GAPDH, at various timepoints following restimulation of WT and cDKO CD4+ T cells relative to Jak1 in IL-2-rested cells at time 0. (**h**) Rate of Jak1 degradation in relative units per hour over 6 h of stimulation. (**i**,**j**) Levels of Jak1, normalized to GAPDH, at various timepoints following rest in the absence of IL-2/TCR, of WT and cDKO CD4+ T cells relative to Jak1 in IL-2-rested cells at time 0. (**j**) As in **g** and **i**, levels of Jak1, normalized to GAPDH, 24 h ±TCR stimulation of WT and cDKO CD4+ T cells relative to Jak1 in IL-2-rested cells at time 0. Data shown in **g**–**j** average ±s.e.m. from two to four biologic replicates in more than three experiments. *P* value calculated by two sample, unpaired *t*-test *P<0.05.

Jak1 was robustly degraded in TCR-stimulated WT cells treated with cycloheximide, a translation inhibitor (Fig. 7a). Ndfip-deficient CD4+ T cells showed significantly less degradation (Fig. 7a,b). We also observed increased phosphorylation of STAT5a/b after stimulation of Ndfip-deficient cells compared with WT cells (Fig. 7c,d). We did not observe any change in Jak1 stability or p-STAT5a/b signal in CD4+ T cells from Ndfip1 cKO mice, suggesting that loss of Ndfip1 alone is not sufficient to affect Jak1 stability and STAT5 activity (Fig. 7a–d). Levels of total STAT5 were not significantly increased in cDKO CD4+ T cells, and STAT5 did not show cycloheximide sensitivity (Fig. 7e,f), consistent with published data^37^. Thus increased levels of p-STAT5 are indicative of increased Jak1 activity in Ndfip-deficient cells.

A balance of protein synthesis and degradation controls Jak1 levels—during T cell activation, Jak1 translation is halted and existing Jak1 is degraded^3^. For both WT and Ndfip-deficient cells, relative levels of Jak1 remaining after stimulation were not altered by cycloheximide (Fig. 7a,b,g). Ndfip-deficient cells showed increased Jak1 after stimulation in either condition, indicating that Jak1 protein synthesis is terminated normally in stimulated cDKO cells. Furthermore, this indicates that Jak1 stability in cDKO T cells is not impacted by new protein synthesis (e.g., production of cytokines). Overall, Jak1 degradation was significantly faster in WT CD4+ T cells compared with cDKO cells (Fig. 7h). Jak1 degradation during the first hours of TCR stimulation was intact in Ndfip-deficient cells; however, further Jak1 degradation was aborted (Supplementary Fig. 7a,b). As with Ndfip1, loss of Ndfip2 alone was not sufficient to affect Jak1 stability during TCR stimulation (Supplementary Fig. 7c).

If Jak1 degradation is promoted by Ndfip-dependent E3 ubiquitin ligases, either directly or indirectly, then unstimulated cells, with ongoing Jak1 synthesis, should show increased Jak1 over time in the absence of Ndfips. In unstimulated cells, we found that Jak1 in WT cells was stable, but in the absence of Ndfip1/Ndfip2 Jak1 levels steadily increased (Fig. 7i). After 24 h in the absence or presence of TCR—after translational recovery occurs in TCR-stimulated cells—Jak1 was significantly increased in cDKO CD4+ T cells as compared with WT cells (Fig. 7j).

### Ndfip-deficient T cells show persistent cytokine signalling

High Jak activity can lead to increased cell viability and proliferation through STAT5 activation and subsequent transcription of STAT5 target genes^38^,^39^,^40^,^41^,^42^. Consistent with this, we observed increased expression, but not aberrant degradation, of the STAT5 target cyclin D2 (refs 31, 43, 44) in stimulated Ndfip1/Ndfip2-deficient CD4+ T cells (Supplementary Fig. 7d). Similarly, surface expression of the IL-2 receptor α (IL-2Rα, aka CD25), another STAT5 target^45^, was increased on stimulated cDKO cells compared with WT CD4+ T cells, even when these cells were co-cultured (Supplementary Fig. 7e,f).

**Figure 8:**
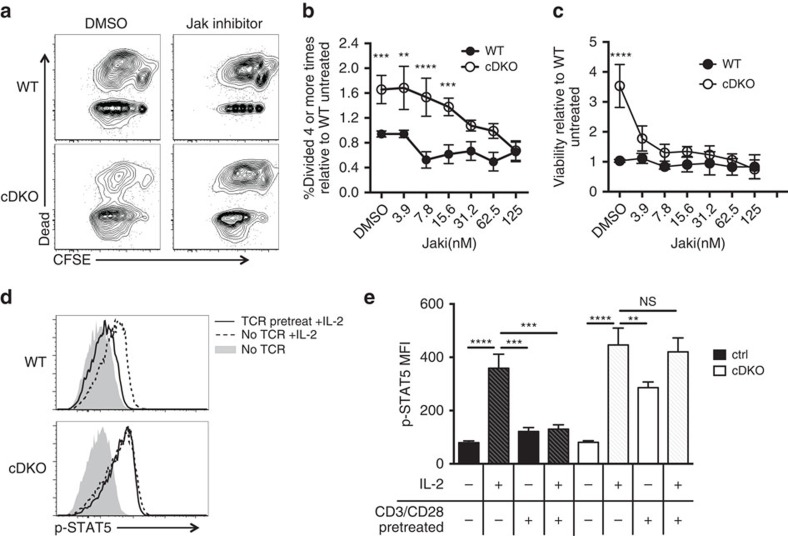
Ndfip-deficient T cells show persistent cytokine signalling. (**a**–**c**) Sorted naive CD4+ T cells from WT and cDKO mice were stimulated with αCD3/CD28 in the presence of Jak inhibitor I (JAKi) and analysed on day 5 by flow cytometry. (**a**) Representative plots of CFSE dilution and viability staining in WT and cDKO CD4+ T cells ±Jak inhibitor (31.2 nM). (**b**,**c**) Quantification of (**b**) percent of cDKO and WT cells divided four or more times relative to DMSO-treated experiment-matched WT cells and (**c**) viability of cDKO and WT CD4+ T cells normalized to DMSO-treated experiment-matched WT cells. Data shown is average ±s.e.m. from six biologic replicates. *P* values calculated by multiple *t*-test with Holm–Sidak correction. (**d**,**e**) p-STAT5 staining in cDKO and WT CD4+ T cells rested in the absence of IL-2 overnight then treated ±αCD3/CD28 beads before addition of IL-2. (**d**) Representative flow cytometry histograms. (**e**) Quantification of p-STAT5 MFI for eight to nine biologic replicates and four independent experiments, showing average ±s.e.m. Patterned bars indicate addition of exogenous IL-2. *P* values calculated by two-way ANOVA with Holm–Sidak test for multiple comparisons. *P<0.05, ^**^P<0.01, ^***^P<0.001, ^****^P<0.0001.

To determine if Jak1 stabilization mediates the observed *in vitro* and *in vivo* hyperactivation and hyperviability of Ndfip-deficient CD4+ T cells, we tested whether Jak inhibition could normalize cDKO levels of proliferation/survival. cDKO CD4+ T cells stimulated *in vitro* in the presence of a Jak inhibitor showed reduced proliferation, down to WT levels, in a dose-dependent manner; WT cell proliferation was responsive to Jak inhibition but did not show dose dependence, suggesting complete Jak inhibition occurred at a lower dose (Fig. 8a,b). CD25 decreased in both WT and cDKO CD4+ T cells in a dose-dependent manner (Supplementary Fig. 7g). Strikingly, cDKO CD4+ T cells showed a robust decline in viability, down to WT levels, when treated with the Jak inhibitor (Fig. 8a,c).

To determine if sensitivity to Jak inhibition was dependent on increased cytokine availability, we co-cultured WT and cDKO CD4+ T cells and treated with the Jak inhibitor. This revealed distinct regulation of viability and proliferation. WT cells cultured with cDKO cells now showed dose-dependent decreases in proliferation in the presence of Jak inhibition, although they still proliferated less than cDKO cells (Supplementary Fig. 8a,b). In contrast, the effect of Jak inhibition on viability was independent of increased cytokine production: the enhanced viability of cDKO cells was lost upon treatment with the Jak inhibitor, which had limited effect on WT cell viability (Supplementary Fig. 8c). Therefore, following activation, the increased survival and proliferative capacity of cDKO CD4+ T cells is due to increased Jak-dependent cytokine signalling. Increased cell viability is independent of increased cytokine production, and can be normalized by Jak inhibition.

During acute TCR signalling, cytokine responsiveness decreases; Jak1 degradation is thought to be required for this desensitization^1^,^2^,^3^. In TCR stimulated CD4+ T cells, decreased induction of STAT5 phosphorylation downstream of the IL-2 receptor limits effector cell expansion by inducing growth arrest and apoptosis^1^. To test whether Jak1 stabilization in Ndfip-deficient T cells allows persistent cytokine signalling, we examined STAT5 phosphorylation after IL-2 treatment in cells that were or were not receiving TCR signals (Fig. 8d,e). In unstimulated cells, p-STAT5 was significantly increased in cDKO CD4+ T cells relative to WT cells on IL-2 treatment, consistent with the increased levels of Jak1 observed in cDKO cells at this time (Fig. 7j). Strikingly, WT CD4+ T cells stimulated through their TCR failed to phosphorylate STAT5 in response to IL-2, while in cDKO cells there was no difference in the p-STAT5 response to IL-2 in the presence or absence of TCR signalling (Fig. 8d,e).

Indeed, cDKO cells exhibited strong STAT5 phosphorylation under TCR stimulation alone, without addition of exogenous IL-2 (Fig. 8e). This was significantly decreased when IL-2 was blocked in TCR stimulated cDKO CD4+ T cell cultures, indicating that cDKO cells persistently signal through IL-2 in an autocrine or juxtacrine fashion while actively signalling through the TCR (Supplementary Fig. 8d). Thus, Ndfip1 and Ndfip2 are required for TCR-induced cytokine desensitization, and, in the absence of Ndfips, stabilization of Jak1 can drive persistent IL-2 signalling to promote cell survival and proliferation.

## Discussion

Fine-tuned control of signalling downstream of the TCR, co-receptors and cytokine receptors is critical in activated T cells. Perturbed signalling can alter T cell function, differentiation, survival and proliferation with life-threatening consequences. One layer of control is degradation of key signal transducers via ubiquitylation to attenuate activating signals. Consistent with this, prior work has detailed the aberrant CD4+ T-cell responses that occur in the absence of the E3 ligase Itch and one of its known activators, Ndfip1 (refs 5, 9, 13, 14, 15, 46).

Here we uncover a role for Ndfip2, another activator of Itch and related ligases, in limiting effector T-cell immunopathology by controlling effector cell expansion. Unlike Ndfip1, Ndfip2 does not impact naive T-cell activation or differentiation; however, together Ndfip2 and Ndfip1 limit the expansion/persistence of effector CD4+ T cells. In previously activated CD4+ T cells, Ndfip1 and Ndfip2 are acting in a coordinated, but perhaps non-redundant, manner to prevent aberrant T cell responses. While expression of both Ndfip1 and Ndfip2 is increased during T-cell stimulation our data indicate distinct magnitude and kinetics, providing one possible explanation for why Ndfip2 knockout mice do not phenocopy mice lacking Ndfip1.

Nothing is known about how Ndfips intrinsically regulate T-effector cells. To investigate this, we developed a proteomics workflow in primary CD4+ T cells for unbiased identification of differential ubiquitylation in primary T cells. Using whole cell proteome analysis, polyubiquitin pulldown and diglycine remnant analysis, we examined differential ubiquitylation in CD4+ T cells sufficient or deficient for Ndfips. Supporting the robustness of this proteomic approach, our analysis identified that, of nine Nedd4 family ligases, Nedd4-2 and Itch are likely enzymatically active during stimulation of effector CD4+ T cells and autoubiquitylated in an Ndfip-dependent manner. This is consistent with previously published work^8^,^13^. Biochemically, we validate that catalytic function of both Itch and Nedd4-2 can be activated or potentiated by Ndfip1 and Ndfip2, indicating that our screen successfully identifies Ndfip-dependent ubiquitylation events.

Using this proteomics approach we identified Jak1 as aberrantly ubiquitylated in the absence of Ndfips. Jak1 has multiple ubiquitylated lysines, and was more stable in stimulated Ndfip-deficient CD4+ T cells. The hyperviability and hyperproliferation of Ndfip-deficient T cells was blocked by Jak inhibition. These inhibitor experiments revealed that, while hyperviability was dependent on increased Jak signalling independent of cytokine production, the role of Jak in proliferation was proportionate to cytokine availability. Consistent with Jak1 stabilization, Ndfip-deficient cells failed to undergo TCR-mediated cytokine non-responsiveness—stimulated Ndfip-deficient cells remained sensitive to IL-2, activating signalling cascades downstream of Jak1 that promote survival and proliferation. These data support that Ndfip-dependent Jak1 degradation plays an important role in limiting cytokine responsiveness during TCR stimulation, although whether this is a direct effect of Ndfip-mediated activation of Jak-targeting E3 ligases remains to be seen.

Notably, in the absence of Ndfips, Jak1 degradation did occur after TCR stimulation, but was limited temporally. Thus, initial Jak1 degradation is Ndfip-independent. If Ndfip-dependent ligases (for example, Nedd4 family ligases) target Jak1 for degradation directly, then one possibility is that these ligases are activated by an Ndfip-independent mechanism downstream of early TCR signalling, allowing them to ubiquitylate Jak1. Activation of these ligases can occur via phosphorylation (Itch, Nedd4, Nedd4-2) and calcium binding (Smurf2, Nedd4, Nedd4-2)^47^,^48^,^49^,^50^,^51^. However, after termination of early signalling events that could relieve ligase autoinhibition in a calcium or phosphorylation-dependent manner, ligase activity may depend on Ndfips. Alternatively, Nedd4 family E3 ligases may be dispensable for early Jak1 degradation. Nevertheless, Ndfip-independent degradation of Jak1 in CD4+ T cells is clearly not sufficient to terminate cytokine signals, as Ndfip-deficient cells fail to undergo TCR-mediated cytokine non-responsiveness.

Single deficiency in either Ndfip1 or Ndfip2 was not sufficient for Jak1 stabilization. These data suggest that Ndfip1 and Ndfip2 compensate for one another in promoting Jak1 degradation in restimulated effector CD4+ T cells. In these previously activated cells, expression of both Ndfips is induced equally well. One intriguing idea is that increased sensitivity of previously activated T cells to TCR restimulation necessitates increased action of Ndfip-dependent E3 ligases, and therefore increased overall expression of Ndfips is required for efficient activation of a larger pool of these E3s. In addition, the sequence of Ndfip1 contains solvent lysine residues in close proximity to its PY motifs, which may be preferentially ubiquitylated when Ndfip1 interacts with and activates Nedd4 family E3 ligases^52^. Ndfip2 does not have these lysines—while this may negatively impact its ability to activate Nedd4 family E3 ligases^52^, it may also extend its half-life, thereby providing a longer lasting, although less efficient, activation signal for Nedd4 E3 ligases.

While our work directly relates to IL-2 promoting survival and proliferation, increased IL-2 signalling has other effects on T cells. Increased phosphorylation of STAT5 downstream of IL-2 receptor signalling promotes T_H_2 differentiation^33^,^53^ and limits T_FH_ differentiation^54^. Previously published substrates of Ndfip-dependent ligases also play roles in promoting T_H_2 differentiation and inhibiting T_FH_ development^5^,^8^,^9^. While degradation of Nedd4 family substrates could promote T_FH_ and limit T_H_2 differentiation, regulation of Jak1 levels, either directly or indirectly by Ndfip-dependent E3 ligases, and downstream cytokine signalling could augment these pathways in a cooperative or synergistic manner.

We propose a model in which Ndfip1 and Ndfip2 work together to limit cytokine signalling by promoting Jak1 degradation (Supplementary Fig. 9). In effector T cells not undergoing TCR stimulation, Ndfip1 and Ndfip2 maintain Jak1 levels in balance with protein synthesis. When T cells are activated via their TCR, Ndfip1 and Ndfip2 enforce a period of cytokine non-responsiveness, by promoting Jak1 degradation, to restrict the effector pool. Promoting TCR-mediated downregulation of cytokine signalling is a previously unknown role for Ndfip1 and Ndfip2 and their cognate E3 ligases. We propose that Ndfip-dependent degradation of Jak1 is critical in determining effector CD4+ T cell numbers that, when disrupted, leads to pathologic accumulation of effector CD4+ T cells.

## Methods

### Mouse strains

*Ndfip1*−/− and *Ndfip1*^fl/fl^CD4Cre+ mice have been described^13^,^20^. *Ndfip1*−/− mice have been backcrossed to the C57BL/6 for more than 10 generations; *Ndfip1*^fl/fl^ mice were derived from insertion of loxP sites into C57BL/6 embryonic stem cells and are maintained on a C57BL/6 background. CD45.1+ (C57BL6.SJL-Ptprca Pepcb/BoyJ) and *Rag1*−/− (B6.129S7-Rag1tm1Mom/J) are maintained in house. *Ndfip2* knockout/GFP knock-in mice were generated by Taconic Biosciences, Inc. (TaconicArtemis) as described in Supplementary Fig. 1 using C57BL/6 embryonic stem cells, and are maintained on C57BL/6 background. Animals were used at 5–7 weeks of age with the exception of the 16-week-old *Ndfip2*−/− and control mice used for phenotypic analysis in Supplementary Fig. 1. *Rag1*−/− hosts for chimera experiments were 8 weeks old. Control animals were *Ndfip1*^fl/fl^ Cre-, *Ndfip1/Ndfip2*+/+, or CD45.1 as appropriate. *Ndfip1/2* DKO mice were generated by breeding *Ndfip2*−/− *Ndfip1*+/− females to *Ndfip2*+/−*Ndfip1*+/− males. cDKO mice were generated by breeding *Ndfip2*−/−*Ndfip1*^fl/fl^CD4Cre- mice to *Ndfip2*−/−*Ndfip1*^fl/+^CD4Cre+ mice. We observed no sex differences in *Ndfip2*−/− or cDKO mice—experiments were performed on both female and male mice using appropriate sex/age-matched controls, and data shown are pooled male and female mice. Mice were maintained in a barrier facility at the Children's Hospital of Philadelphia. All procedures were approved by the Institutional Care and Use Committee of the Children's Hospital of Philadelphia. *Ndfip2* KO/KI alleles were detected using the following primers on genomic DNA: WT_F 5′-CCCTGTGCCACCTCCGTACAGTG-3′; WT_R 5′-GCTGAGGCAGTGCGCAGACTTAC-3′; KO/KI_F 5′-CTTCAAGCAGACCTACAGCAAG-3′; KO/KI_R 5′-CCTGTTATCCCTAGCGTAACG-3′. The FLP transgene was detected using the following PCR primers on genomic DNA: FLP_F 5′-GACAAGCGTTAGTAGGCACAT-3′; FLP_R 5′-GGCAGAAGCACGCTTATCG-3′. All animals used in experiments described were FLP negative. Genotyping primers for *Ndfip1* knockout alleles, *Ndfip1* flox alleles and CD4Cre have been described previously.

### Flow cytometry and immunoblot antibodies

All flow cytometry antibodies were used at 1:200 unless otherwise noted. The following flow cytometry antibodies were purchased from Biolegend: CD4 (GK1.5), CD8α (53-6.7), CD44 (IM7), IL-17A (TC11-18H10.1), CD3ɛ (17A2), B220 (RA3-6B2), CD45.2 (104) and CD45.1 (A20). Antibodies against IFNγ (XMG1.2) and Ki67 (B56) were purchased from BD Biosciences. The remaining antibodies for flow cytometry were purchased from eBioscience: IgM (II/41), CD25 (PC61.5), CD62L (MEL-14), GATA3 (TWAJ), IL-4 (11B11), IgM (II/41), T-bet (4B10), FoxP3-biotin (FJK-16s, 1:150), CD93/AA4.1 (AA4.1). Biotinylated αFoxP3 was detected with fluorophore-conjugated streptavidin (S32354 Invitrogen, 1:500). αp-STAT5a/b for flow cytometry was purchased from BD (p Y694, 47). The following primary antibodies were used for immunoblotting: rabbit αJak1 (3332; Cell Signaling Technologies, 1:500), monoclonal rabbit αJak2 (D2E12, Cell Signaling Technologies, 1:500), rabbit αp-STAT5a/b (C11C5; Cell Signaling Technologies, 1:500), monoclonal mouse αtubulin (B-5-1-2; Sigma, 1:1,000), monoclonal mouse αgapdh (mab374; Millipore, 1:2,000), monoclonal mouse αItch (32; BD Biosciences, 1:500), rabbit αNedd4-2 (4013, Cell Signaling Technologies, 1:500), rabbit αCyclin D2 (M-20, Santa Cruz Biotechnology, 1:1,000) and monoclonal rabbit αSTAT5a (E289, Abcam, 1:500).

### GST pulldown

Ndfip cytoplasmic domain constructs were prepared as previously described^29^. Plasmids were transformed into BL21(DE3) *Escherichia coli* and purified by glutathione sepharose 4B (GE Life Sciences). WT and *Ndfip1/Ndfip2* knockout CD4+ T cells were cultured as described below. Rested cells were restimulated for 4 h with PMA/ionomycin as described below, lysed in 1% NP40 (1% NP40, 150 mM NaCl, 50 mM TrisHCl with phosphatase and protease inhibitors), precleared with GST, and then incubated with 10 μg recombinant Ndfip1, Ndfip2 or GST protein. Protein was collected on glutathione sepharose beads, and eluted by addition of 4 × Laemmli sample buffer with boiling. Bound protein was analysed by SDS–polyacrylamide gel electrophoresis (SDS–PAGE) and western blotting as described below.

### Polyubiquitylation assay and ubiquitin charging assay

Polyubiquitylation was monitored using a homogeneous E3 ligase TR-FRET assay (Progenra, Inc.) as described^21^. Briefly, ubiquitylation Mix containing Itch or Nedd4L was combined with varying doses of Ndfip1/2 and HTRF Detection Mix. TR-FRET was monitored in real time using a PerkinElmer Envision plate reader (Ex 340 nm; Em1 520 nm; Em2 480 nm); TR-FRET ratio was calculated as Em_520_/Em_480_. Ubiquitin charging assay and expression of recombinant Itch were described by Riling *et al.*^29^: recombinant E2, E1, and ubiquitin were incubated on ice in the presence of ATP and magnesium to generate pre-charged ubiquitin∼E2 conjugates. This charging reaction was quenched by addition of EDTA, after which E3 ligase (Itch) was introduced to the reaction in the presence or absence of GST-Ndfip cytoplasmic domains. The reaction proceeded for 5 s before addition of reducing sample buffer. Ubiquitylated products were resolved by SDS-PAGE and western blotting as described below.

### Tissue processing and flow cytometry

Spleens, thymii and lymph nodes were harvested and mashed through 70 μM nylon filters using cold Hanks' Balanced Salt Solution (HBSS). Spleens were red blood cell lysed using ammonium-chloride-potassium (ACK) lysis buffer, washed, filtered again and resuspended in cold HBSS. Bone marrow was isolated by flushing femur and tibia with cold HBSS, red blood cell lysing and passing through a 70 μM filter. Lungs were flushed with cold PBS immediately after euthanasia and processed as described^14^. The cells were then sorted; CD4 enriched; stimulated for 4 h with PMA (30 ng ml^−1^, Calbiochem), ionomycin (1 μM, Abcam) and Brefeldin A (1 μg ml^−1^, Sigma) for intracellular cytokine staining; or directly stained for flow cytometry. Cells were washed in serum-free HBSS or PBS, stained with live/dead fixable blue dead cell stain (L-23105; Invitrogen), Fc blocked (αCD16/32, 2.4G2; BD Biosciences) and stained with the appropriate antibody mixtures. After staining for 25 min at 4 °C, samples were fixed using the FoxP3 fix/perm kit (eBioscience). Intracellular staining was done for 1 h at 4 °C. In Supplementary Fig. 2, *Ndfip2*+/− (GFP+) cells were analysed directly, without fixation, for expression of GFP. In Fig. 8 and Supplementary Fig. 8d, bulk isolated CD4+ T cells were stimulated and rested as described below, rested one additional night in the absence of IL-2 and then restimulated with mouse T cell activator beads (Invitrogen) at a 1:1 cell:bead ratio for 5.5 h before treatment with 50 U ml^−1^ IL-2 for 20 min. Samples were immediately fixed with 2% PFA, permeabilized in methanol and then stained for p-STAT5 and surface markers together. Samples were acquired on an LSRFortessa (BD Biosciences), and analysed using FlowJo version 9.8 (Flowjo LLC). Events analysed were singlets (FSC-A × FSC-H), live (viability dye negative) and within the lymphocyte gate (FSC-A × SSC-A).

### T-cell isolation and culture

Total CD4+ T cells were isolated from spleen and lymphnode by negative selection using rat αmouse CD8α (2.43; ATCC, 2.5 ml hybridoma supernatant per animal) and rat αmouse I-A/I-E (M5/114.15.2; Biolegend, 5 μl per animal). Cell suspensions were incubated with the antibodies in complete DMEM for 1 h with end-over-end rotation at 4 °C, washed and incubated with Biomag goat αrat immunoglobulin-G magnetic beads (Qiagen) for 15 min with end-over-end rotation at room temperature; unbound cells were collected using a Dynal magnet (Invitrogen). Purity after isolation and subsequent culture was >85% CD4+CD3+ T cells. To sort naive CD4+ T cells, single cell suspensions of spleen and lymphnode were stained with antibodies against CD4, CD8α, CD44, CD25 and CD62L. Cells were filtered through 35 μM filter cap polystyrene FACS tube (BD Biosciences) and sorted under high speed on a MoFlow Astrios (Beckman Coulter) or a FACs Aria (BD Biosciences). Naive cells were identified as CD4+CD62L^high^CD44-CD25-. Cells were resuspended at 1 × 10^6^ cells ml^−1^ before stimulation with 5 μg ml^−1^ plate-bound αCD3 (145-2C11; Biolegend) and αCD28 (37.51; Biolegend) for the times indicated. All cultured cells, unless otherwise noted, were cultured at 10% CO_2_ in DMEM (Mediatech) supplemented with 10% foetal calf serum (Atlanta Biologicals), 1% pen/strep (Invitrogen), 1% Glutamax (Invitrogen) and 0.12 mM betamercaptoethanol (Sigma). Unless otherwise noted, after 4 days of stimulation, bulk isolated CD4+ T cells were rested/expanded in 50 U ml^−1^ recombinant human IL-2 (obtained through the AIDS Research and Reference Reagent Program, Division of AIDS, National Institute of Allergy and Infectious Diseases, National Institutes of Health) for 3 days, and then restimulated. Naive cells were stimulated for 5 days. In Supplementary Fig. 3, naive CD4+ T cells were polarized: Th1: αIL-4 (20 μg ml^−1^, 11B11; BioLegend), IL-12 (10 ng ml^−1^ Peprotech), IL-2 (50 U ml^−1^); Th2: αIL-12/23p40 (20 μg ml^−1^, C17.8; Biolegend), αIFNγ (20 μg ml^−1^, XMG1.2; Biolegend), IL-4 (7.5 ng ml^−1^ Peprotech), IL-2 (50 U ml^−1^); iTreg: IL-2 (50 U ml^−1^), TGFβ (5 ng ml^−1^ Peprotech); Th17: IL-23 (50 ng ml^−1^ R&D Systems), IL-6 (20 ng ml^−1^ eBioscience), TGFβ (0.5 ng ml^−1^ Peprotech), IL-1β (20 ng nl^−1^ Peprotech). On day 5 of culture, polarized cells were restimulated with PMA (30 ng ml^−1^), ionomycin (1 μM) and brefeldin A (1 μg ml^−1^) for intracellular cytokine staining or stained directly. For CFSE labelling (Fig. 8 and Supplementary Fig. 8), cells were washed in PBS, resuspended at <1 × 10^7^ ml^−1^ in 500 μl room temperature PBS and mixed at a 1:1 ratio with CFSE (2.5 μM final concentration, Life Technologies) in PBS for 3 min with constant agitation. Labelling was quenched with FCS and cold DMEM. In Supplementary Fig. 2, Ndfip2+/− (GFP+) cells were labelled with ef670 cell proliferation dye prior to stimulation according to the manufacturer's instructions (eBioscience). For cocultures (Supplementary Figs 5, 7 and 8), naive CD4+ T cells were sorted from WT CD45.1 and cDKO (CD45.2) spleen and lymphnodes. Cells were mixed in a 1:1 ratio and then CFSE labelled before being stimulated. In Supplementary Fig. 8d, mouse IL-2 signalling was inhibited with 20 μg ml^−1^ αIL-2 blocking antibody (JES6-1A12, Biolegend).

### Western blotting and cycloheximide treatment

Bulk isolated CD4+ T cells were resuspended at 1 × 10^6^ cells ml^−1^ in complete DMEM and stimulated with αCD3/CD28 for 4 days. Cells were rested and expanded in recombinant human IL-2 at 50 U ml^−1^ (3–4 days) prior to restimulation with plate-bound αCD3/CD28 for the indicated periods. Where noted, cycloheximide (Sigma) was added at 10 μg ml^−1^ after 2 h of restimulation, and cells were incubated for an additional 2–4 h. Cells were collected with cold PBS, pelleted and lysed for 15 min on ice with digitonin lysis buffer^55^. Lysates were then centrifuged at 4,500 r.c.f. for 4 min, and separated from the pellet. Digitonin insoluble pellet was subsequently lysed in 1% SDS, 10 mM tris, 20 μg ml^−1^ DNase I and Roche complete inhibitor tablet with boiling. A concentration of 4 × Laemmli buffer was then added to 1 × final concentration and samples were boiled for 5 min. Lysates were loaded by cell number equivalents on pre-cast Criterion Tris-HCL gels (Biorad) and subjected to SDS–PAGE. Protein was transferred to polyvinylidene difluoride (Millipore) using a semi-dry transfer apparatus. Membrane was blocked in Roche blocking reagent (11096176001). Protein was visualized on an Odyssey imager (LICOR) using AF680 goat αrabbit (Invitrogen), IRdye 800 donkey αmouse (LICOR) and IRdye 800 donkey αgoat (LICOR) secondary antibodies. Bands were quantified using Image Studio Lite (LICOR). Western blots have been cropped for clarity in the figures. Uncropped blots with size markers can be found in Supplementary Figs 10–13.

### Quantitative PCR

qPCR was performed as previously described^14^. In brief, 10 ng of complementary DNA was added to TaqMan Gene Expression Master Mix and TaqMan Gene Expression primer/probe mix specific for Ndfip1 or Ndfip2 (Applied Biosystems) for a final reaction volume of 20 μl. qPCR was performed using an Applied Biosystems 7500 Real-Time PCR system. Each sample was assayed in triplicate along with the endogenous control (actin). ACTB primer/probe (4352933E) was obtained from Applied Biosystems. Ndfip1 and Ndfip2 custom primer and probe sequences are as follows: Ndfip1_F 5′-GCTCCTCCACCATACAGCAGC-3′; Ndfip1_R 5′-CGATGGGGGCTTTGGAAATCCAG-3′; Ndfip1 Taqman MGB probe 5′-TTTGGAAATCCAGATTCATCTTTG-3′; Ndfip2_F 5′-AGCAGCATCACTGTGGAAGCT-3′; Ndfip2_R 5′-GGCACAGGGTAAAACTCACTATACAC-3′; Ndfip2 Taqman MGB probe 5′-CTACCACTTCAGATACTG-3′.

### Foetal liver chimeras

Foetal liver from WT (CD45.1+), *Ndfip1*−/− (CD45.2+), *Ndfip2*−/− (CD45.2) and Ndfip1/2 DKO (CD45.2) embryos was processed into a single-cell suspension by mashing through a 35-μm filter. Embryos were genotyped at the time of harvest. Cells were resuspended in freezing media (90% FCS, 10% DMSO) and kept at −80 °C until used. Thawed cells were washed, counted, mixed 1:1 CD45.1:CD45.2, resuspended in sterile PBS and injected i.v. into sublethally irradiated 6-week-old Rag1−/− recipients, 1 × 10^6^ cells per mouse. Chimeras were weighed twice weekly and analysed ∼6 weeks after transfer, when inflammation and weight loss were observed.

### Histology

Skin, oesophagus and distal colon were dissected and fixed in 10% formalin for at least 24 h. Lung was perfused with formalin. All organs were then embedded in paraffin, sectioned and stained with H&E.

### T-cell transfer colitis

Naive CD4+ T cells from WT, *Ndfip2*−/−, *Ndfip1*^*fl/fl*^CD4Cre+ and Ndfip2−/−, Ndfip1fl/flCD4Cre+ mice were sorted from spleens and lymph nodes as described above. Cells were resuspended at 5 × 10^6^ ml^−1^ in sterile PBS and 0.5 × 10^6^ cells were injected intraperiotneally into 6–8-week-old *Rag1*−/− mice. Mice were weighed every 2–3 days. Recipients were co-housed. Clean cage bedding was mixed with dirty bedding such that cages were only fully changed every 2 weeks. Mice were killed when recipients showed weight loss of 30% starting weight. At the time of killing, mice were weighed, spleens were weighed and processed for flow cytometry as described above, colons were measured for contraction and distal colon sections were fixed for histology. Crypt depth was measured at three different points/slide on H&E-stained colon sections, and averaged/animal.

### TUBE affinity purification and whole proteome analysis

Bulk isolated CD4+ T cells were isolated as described above. Dialysed foetal calf serum was used in SILAC DMEM/SILAC RPMI during isolation. Cells were stimulated in SILAC media with plate-bound αCD3/CD28 for 3 days. SILAC media components are as follows: SILAC RPMI (Hyclone), C13 or C12 L-arginine:HCl and L-lysine:2HCl (Cambridge Isotope Laboratory), 1% MEM NEAA (Invitrogen), 1% sodium pyruvate (Invitrogen), 1% Glutamax (Invitrogen), 1% pen/strep (Invitrogen), 10% Dialysed FCS (Life Technologies), 2% HEPES (Fisher Scientific) and 0.12 mM betamercaptoethanol (Sigma). After stimulation, cells were rested in IL-2 for 3 days. Cells were restimulated at 4 × 10^6^ ml^−1^ SILAC media using PMA (30 ng ml^−1^) and ionomycin (1 μM) for 4 h. MG132 (10 μM ml^−1^; Calbiochem) and chloroquine (50 μM ml^−1^; Sigma) were added during the final 2 h of stimulation. Cells pellets were lysed using the recommended lysis buffer for product UM604 from Lifesensors. Protein was quantified by BCA. Approximately 30 μg of ‘input' unmixed lysate was mixed 1:1 with 4 × Laemmli sample buffer and saved for whole proteome analysis. Equal amount of heavy and light lysate was mixed (1.2 mg of each) and diluted 1:10 in lysis buffer with no detergent. Samples were rotated at 4 ° with 50 μg biotinylated pan-TUBE (UM301; Lifesensors) for 2 h. A volume of 250 μl per sample Dynabeads MyOne Streptavidin c1 (Invitrogen) was washed and added to samples, which were rotated for an additional 2 h at 4 °. Beads were collected and washed in cold PBS three times. Samples were eluted using 1 × Laemmli sample buffer and boiling, and were stored at −80 °C.

### In-gel digest

TUBE-enriched SILAC lysates or whole proteome ‘input' samples were run ∼2 cm past the stacking gel in 10% Criterion pre-cast Tris-HCL gels (Bio-Rad). Gels were fixed overnight and stained briefly with Coomassie blue. Each lane of the Coomassie-stained gel was divided into ten 2 mm × 9 mm ‘pixels,' each cut into 1 mm^3^ cubes^56^. They were destained with 50% methanol/1.25% acetic acid, reduced with 5 mM dithiothreitol (Thermo) and alkylated with 40 mM iodoacetamide (Sigma). Gel pieces were then washed with 20 mM ammonium bicarbonate (Sigma) and dehydrated with acetonitrile (Fisher). Trypsin (5 ng μl^−1^ in 20 mM ammonium bicarbonate, Promega) was added to the gel pieces and proteolysis was allowed to proceed overnight at 37 °C. Peptides were extracted with 0.3% triflouroacetic acid (J.T. Baker), followed by 50% acetonitrile. Extracts were combined and the volume was reduced by vacuum centrifugation.

### K-ɛ-GG peptide immunoprecipitation

Bulk CD4+ T cells were stimulated and expanded in IL-2, as above, prior to restimulation for 4 h with PMA/ionomycin (30 ng/1 μM) including MG132 (10 μM ml^−1^ Calbiochem) and chloroquine (50 μM ml^−1^) for 2 h, or restimulation for 4 h with mouse T cell activator beads (Invitrogen) at a 3:1 cell:bead ratio in the absence of inhibitors. Cell pellets were lysed in urea buffer, protein concentration was measured via micro BCA assay (Thermo), and peptides were prepared and immunoprecipitated as described^25^,^57^. Approximately 250 × 10^6^ total CD4+ T cells at the start of stimulation yielded ∼3 mg of total peptide. Peptides were off-line basic reverse phase fractionated and recombined noncontiguously into three fractions of ∼1 mg peptide per fraction for immunoprecipitation. PTMscan ubiquitin remnant antibody, noncovalently conjugated to beads (Cell Signaling Technologies) was crosslinked as described^25^; crosslinking was validated by SDS–PAGE. 31 ug of crosslinked antibody was used for each 1 mg peptide fraction. The following modifications were made to the published protocol: step 6: alkylation was done with 20 mM IAM; step 10: peptides were acidified by 1% formic acid (final concentration); step 37: beads were washed 2 × with IAP, followed by washing 2 × with IAP plus 0.05% RapiGest SF surfactant (Waters), followed washing 3 × with PBS; step 41: the eluted K-ɛ-GG peptides were desalted via Oasis HLB uElution plate 30 μM (Waters). All mass spec samples were prepared in 0.1% TFA/water and analysed as described below. After LC-MS/MS analysis, >50% of peptides were modified.

### LC-MS/MS

Tryptic digests were analysed by LC-MS/MS on a hybrid LTQ Orbitrap Elite mass spectrometer (Thermofisher Scientific) coupled with a nanoLC Ultra (Eksigent). Peptides were separated by reverse phase (RP)-HPLC on a nanocapillary column, 75 μM ID × 15 cm Reprosil-pur 3 μM (Dr Maisch, Germany) in a Nanoflex chip system (Eksigent). Mobile phase A consisted of 0.1% formic acid (Thermofisher Scientific) and mobile phase B of 0.1% formic acid/80% acetonitrile. Peptides were eluted into the mass spectrometer at 300 nl min^−1^ with each RP-LC run comprising a 90-min gradient from 10 to 25% B in 65 min, 25–40% B in 25 min, followed by column re-equilibration. The mass spectrometer was set to repetitively scan *m*/*z* from 300 to 1,800 (*R*=240,000 for LTQ-Orbitrap Elite) followed by data-dependent MS/MS scans on the 20 most abundant ions, with a minimum signal of 1,500, dynamic exclusion with a repeat count of 1, repeat duration of 30 s, exclusion size of 500 and duration of 60 s, isolation width of 2.0, normalized collision energy of 33, and waveform injection and dynamic exclusion enabled. FTMS full scan AGC target value was 1e6, while MSn AGC was 1e4, respectively. FTMS full scan maximum fill time was 500 ms, while ion trap MSn fill time was 50 ms; microscans were set at one. FT preview mode; charge state screening, and monoisotopic precursor selection were all enabled with rejection of unassigned and 1+ charge states.

### Data analysis

TUBE–SILAC data were analysed using Maxquant verison 1.5.0.30 using the Uniprot complete mouse reference proteome, including isoforms (updated Sept 19, 2013) and common laboratory contaminants with a minimum peptide length of 6 amino acids and a peptide and protein false discovery of 1%. The four biologic replicates for TUBE–SILAC experiments were analysed together, with match between runs and requantify turned on. SILAC ratios were calculated in MaxQuant. Whole proteomes were analysed together in MaxQuant version 1.5.1.2, using the Uniprot complete mouse reference proteome (updated 18 August 2014) and common lab contaminants with a minimum peptide length of 6 amino acids and 1% false discovery rate; requantify and match between runs were turned off. Unique peptide counts were used to generate semiquantitative ratios representing relative protein abundance, DKO/WT. Proteins analysed had a minimum of three unique peptides identified. In the case of missing values, 0 count was defined as 1. To correct TUBE–SILAC ratios for input proteome ratios for each protein, the log2 transformed average unenriched DKO/WT input ratio was added to the log2 transformed normalized TUBE–SILAC ratio (WT/DKO). TUBE–SILAC ratios were corrected for each of four biologic replicates, including one isotope swap. K-ɛ-GG data was searched using SEQUEST, and visualized in Scaffold Viewer version 4.3.4 (Proteome Software, Inc.). Proteins with at least one modified peptide were considered for further analysis. The two biologic replicates of αCD3/28 stimulated CD4+ T cells in the absence of inhibitor were each analysed twice by LC-MS/MS, and data from these technical replicates were summed for further analysis. In Fig. 5, the heatmap was generated by one-matrix CIM with columns and rows clustered by average linkage and Euclidean distance with quantile bins^58^. The area proportional Venn diagram was generated with eulerAPE version 3.0 (ref. 59).

### Statistical analysis

Data were graphed and analysed for statistical significance in Prism version 6 (Graphpad Software, Inc), or Excel (Microsoft). The following statistical tests were used as appropriate and as noted in the figure legends: *T*-test, one-way ANOVA, two-way ANOVA, repeated measures ANOVA, one phase exponential decay fit. All data are shown as average ±s.e.m., with a cutoff of *P*<0.05 for statistical significance: *P<0.05, ^**^P<0.01, ^***^P<0.001, ^****^P<0.0001.

## Additional information

**How to cite this article:** O'Leary, C. E. *et al.* Ndfip-mediated degradation of Jak1 tunes cytokine signalling to limit expansion of CD4+ effector T cells. *Nat. Commun.* 7:11226 doi: 10.1038/ncomms11226 (2016).

## Supplementary Material

Supplementary Information Supplementary Figures 1-13 and Supplementary Table 1

Supplementary Data 1 SILAC ratios obtained following TUBE affinity purification withcorresponding whole cell proteome abundance in WT and DKO CD4+ T cells. Limits on SILAC data: 3 or 4 ratios (out of 4 experiments). Limits on while proteome: 3+ unique peptides identified over 4 runs (2 WT, 2 DKO).

Supplementary Data 2 Proteins identified with at least one spectral count containing a K-ɛ-GG motifafter K-ɛ-GG immunoprecipitation in CD4+ WT T cells stimulated as indicated.

Supplementary Data 3 Proteins identified as in dataset 1 that were also found to have at least one modified lysine residue (dataset 2). Hierarchical clustering performed using CIMminer.

Supplementary Data 4 Top proteins predicted to have differential ubiquitylation in WT and DKO CD4+ T cells. Defined as having: SILAC ratios in 4/4 experiments, corrected ratios indicating greater ubiquitylation in WT cells (positive log2 ratios), reproducibleobservation of K-e-GG motif peptide (3/3 experiments, with 5+ total spectral counts).
