# Supplementary material for: Ndfip-mediated degradation of Jak1 tunes cytokine signalling to limit expansion of CD4+ effector T cells
Source: Nat Commun. 2016 Apr 18;7:11226. doi: 10.1038/ncomms11226 (PMC4837450; doi:10.1038/ncomms11226)
Supplement: Supplementary Information — Supplementary Figures 1-13 and Supplementary Table 1 [file ncomms11226-s1.pdf]

Supplementary Figure 1

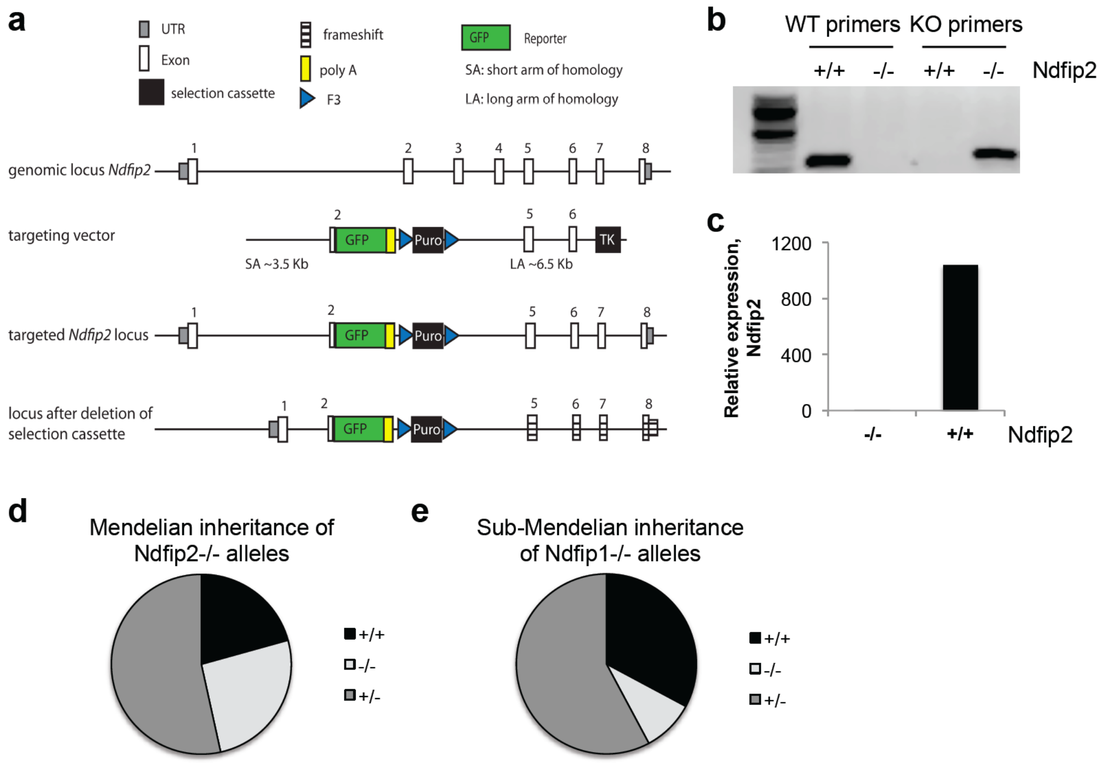

Supplementary Figure 1. Successful generation of Ndfip2 knockout/GFP knock-in mouse

(**a**) Targeting vector used for insertion of GFP into exon 2 of the *Ndfip2* locus. Gene targeting resulted in the insertion of GFP into exon 2, partial deletion of exon 2, and deletion of exons 3 and 4. Downstream exons are out of frame. (**b,c**) Homozygous gene targeted mice were identified by PCR (**b**), and (**c**) further analyzed for *Ndfip2* expression by qPCR. (**d**) PCR genotyping results of 58 pups from heterozygous Ndfip2+/- breeders. (**e**) PCR genotyping results of 64 pups from heterozygous Ndfip1+/- breeders.

Supplementary Figure 2

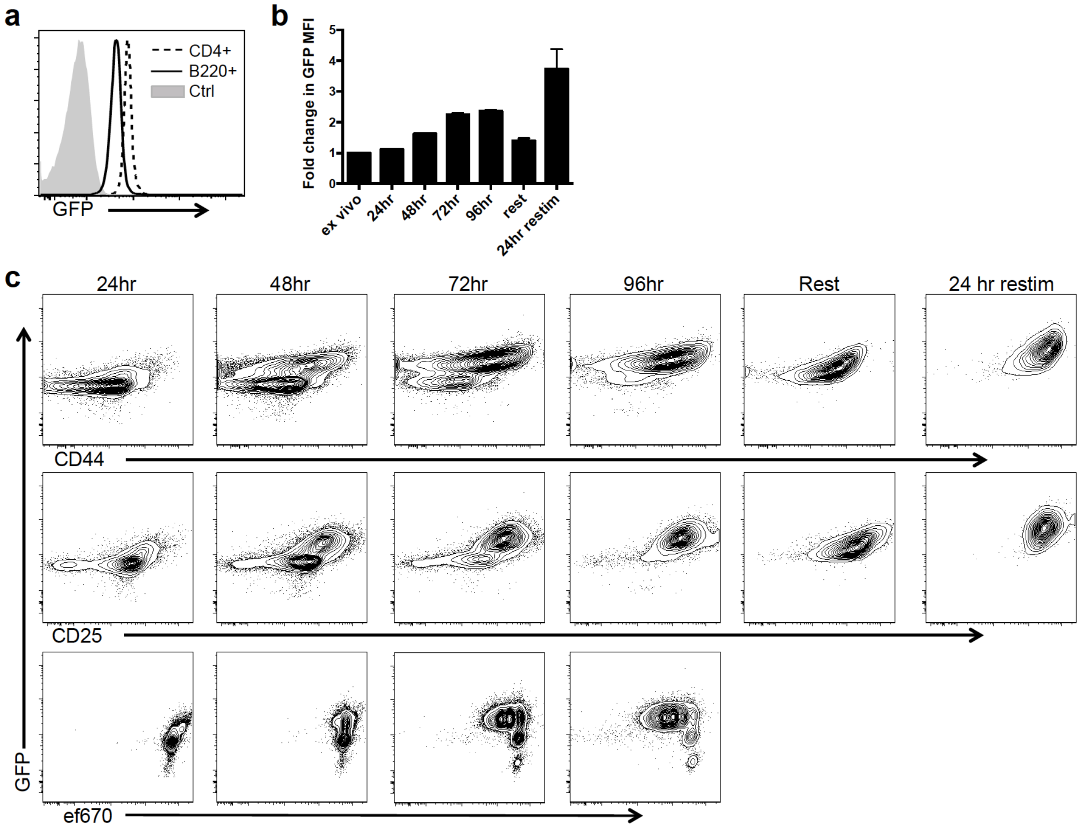

Supplementary Figure 2. Analysis of Ndfip2 GFP reporter expression.
(**a**) GFP expression in *Ndfip2*+/- cell was analyzed using flow cytometry on *ex vivo* splenic B and T cells. (**b,c**) *Ndfip2*+/- CD4+ T cells were labeled with a cell proliferation dye (ef670) and stimulated with plate-bound αCD3/CD28 for the indicated time periods. GFP, CD44, and CD25 expression, and ef670 dilution, were assessed daily by flow cytometry. (**b**) Quantification of GFP MFI relative to *ex vivo* GFP expression in Ndfip2+/- splenic CD4+ T cells, average shown +/- SEM, two biologic replicates. (**c**) Representative flow plots of GFP expression relative to expression of CD44, CD25, and dilution of proliferation dye. Previously gated on CD4+ live singlets.

Supplementary Figure 3

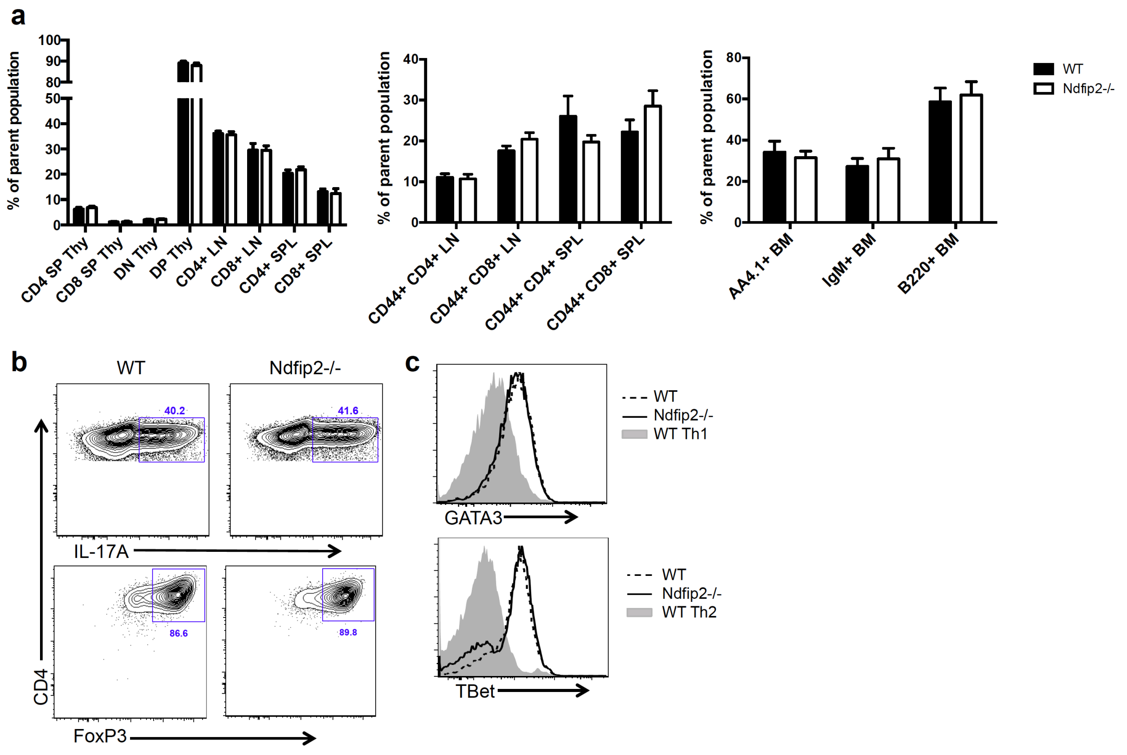

Supplementary Figure 3. No signs of inflammation in Ndfip2-/- mice.
(**a**) Quantification of T cell percentages in thymus, lymph nodes and spleen; % CD44+ CD4 and CD44+ CD8 T cells in spleen and lymph nodes; B cell percentages in bone marrow. Averages shown +/- SEM n=5-6 16 week old Ndfip2-/- mice and age-matched controls. No significant differences by paired t-test. (**b,c**) Naïve CD4+ T cells were sorted from 6 week old Ndfip2-/- and WT mice and stimulated with plate-bound αCD3/CD28 for 5 days in the presence of iTreg (**b**), T_H17 (**b**), T_H1 (**c**), or T_H2 (**c**) polarizing cytokine. On day 5, expression of FoxP3, T-Bet and GATA3 was examined by intracellular staining. Production of IL-17A (T_H17) was analyzed after PMA/ionomycin stimulation in the presence of brefeldin A. Flow plots representative of at least 3 experiments.

Supplementary Figure 4

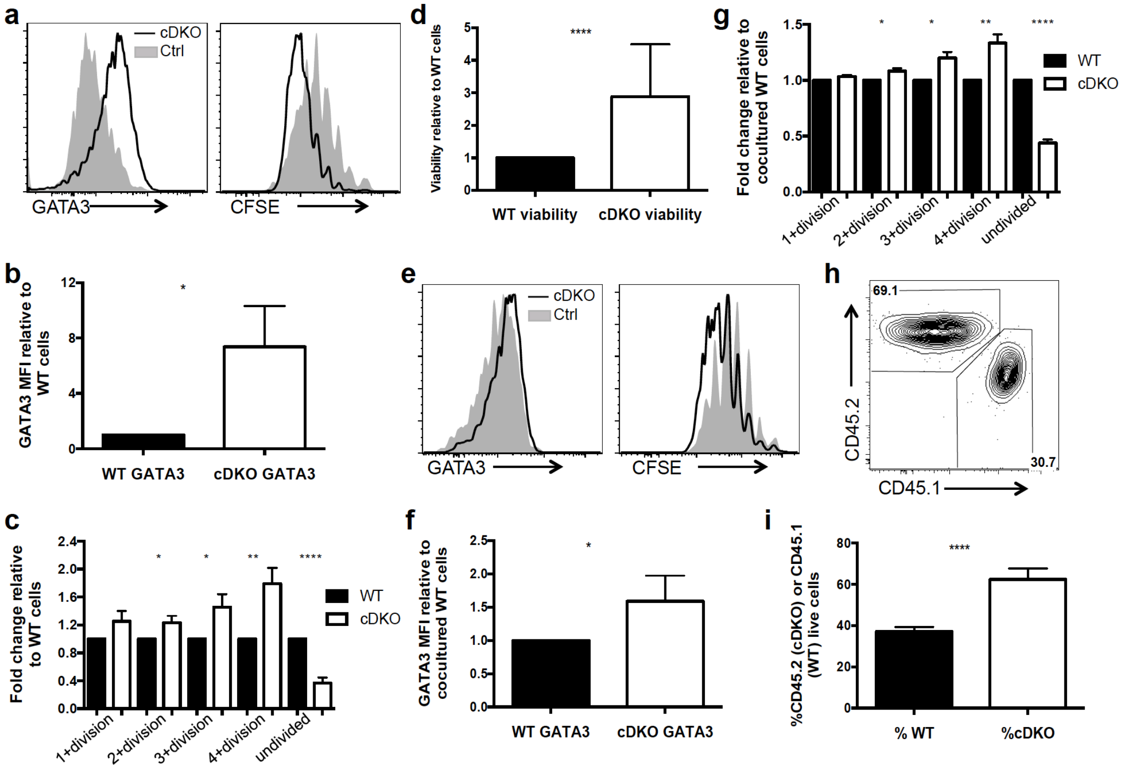

Supplementary Figure 4. Ndfip1/Ndfip2 deficient T cells outcompete WT cells *in vitro*

(**a-d**) Naïve CD4+ T cells were sorted from age-matched cDKO and control mice, CFSE labeled, and stimulated for 5 days with plate-bound αCD3/CD28. (**a**) GATA3 and CFSE dilution were evaluated on day 5 by flow cytometry. Previously gated on live singlets, CD4+. (**c,d**) Quantification of (**a**). (**e**) Viability was determined by % of cells negative for viability dye multipled by % of cells within lymphocyte gate. (**f-i**) Naïve CD4+ T cells were sorted from age-matched cDKO and congenic CD45.1 WT mice, mixed in a 1:1 ratio, CFSE labeled, and stimulated for 5 days with plate-bound αCD3/CD28. (**f**) GATA3 and CFSE dilution were evaluated on day 5 by flow cytometry. Previously gated on live singlets, CD4+, CD45.2+ or CD45.1+. (**g,h**) Quantification of (**f**). (**g**) Representative flow plot showing %CD45.2+ and %CD45.1+ live CD4+ cells on day 5. Percent CD45.2 or CD45.1 quantified in (**h**). All quantifications except (**g**) done relative to experiment matched WT cells, average shown +/-SEM, (**a-d**) n=14-17, (**f-i**) n=6-7. P values calculated by one sample t-test against the normalized WT value of 1, except for (**g**), in which a paired t-test was used. * $p<0.05$, ** $p<0.01$, ***$p<0.001$, ****$p<0.0001$.

Supplementary Figure 5

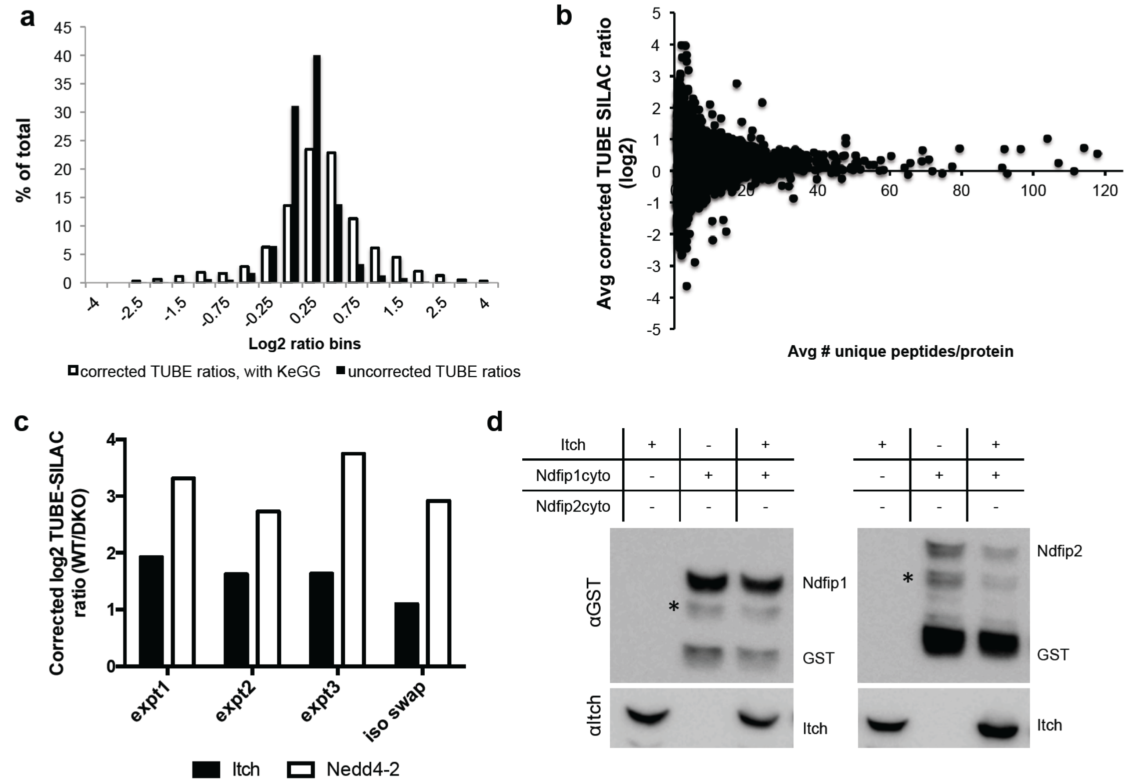

Supplementary Figure 5. Unmodified protein SILAC-TUBE ratios compared to corrected protein SILAC-TUBE ratios and Nedd4 family E3 ligase analysis.

(**a**) Uncorrected log2 transformed SILAC-TUBE ratios, averaged over 4 biologic replicates, compared to corrected SILAC-TUBE ratios (log2 transformed) averaged over 4 biologic replicates and limited to proteins with KGG peptides. Median uncorrected log2 SILAC-TUBE ratio observed as WT/DKO=0.054 over 2376 proteins, median corrected ratio WT/DKO=0.23 over 2376 proteins, median corrected ratio limited to KGG proteins=0.239 over 925 proteins. (**b**) Average log2 transformed corrected SILAC-TUBE ratios plotted by average number unique peptides observed/protein over 4 whole proteome experiments. (**c**) Corrected SILAC-TUBE ratios for Nedd4L and Itch across 4 biological replicates. Log2 transformed ratios (WT/DKO). (**d**) Western blot analysis for purity of recombinant Ndfip-GST proteins and Itch utilized in TR-FRET ubiquitylation assay (**Fig. 6c**). * Indicates Ndfip-GST cleavage product.

Supplementary Figure 6

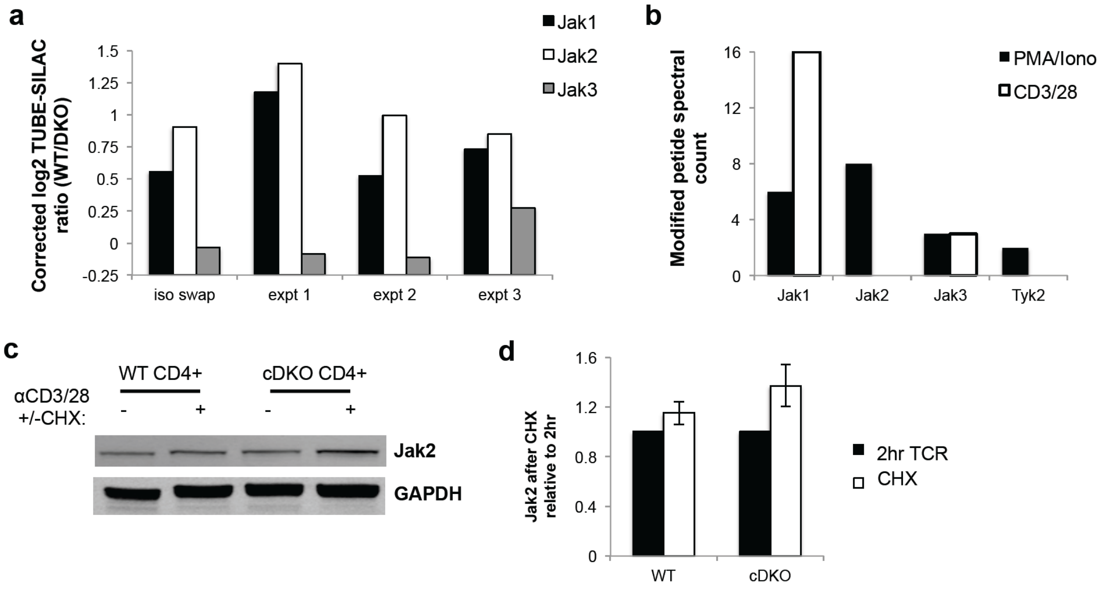

Supplementary Figure 6. Jak family proteomics
(**a**) Corrected SILAC-TUBE ratios for Jak family members across 4 biological replicates. Log2 transformed ratios (WT/DKO). Tyk2 was not reproducibly identified after TUBE enrichment. (**b**) Sum of Jak family protein observed peptides with diglycine remnant on lysine (total spectral counts of modified peptides) after three individual K-ε-GG IP experiments using two distinct stimuli on WT CD4+ T cells: PMA/ionomycin stimulation done for 4 hrs with 2hrs addition of 10μM MG132, 50μM chloroquine; or αCD3/CD28 stimulation done for 4hrs with antibody-coated beads, no inhibitors, sum of 2 immunoprecipitation experiments with double injections. (**c,d**) Immunoblotting of restimulated WT and cDKO CD4+ T cells. Cycloheximide was added 2hrs after αCD3/CD28 stimulation; cells were then incubated an additional 2hrs. (**d**) Level of Jak2 was normalized to GAPDH. The stability of Jak2 was determined by normalizing the relative Jak2 remaining after stimulation to the amount of Jak2 at 2hrs. Data shown are average +/- SEM from 3 biologic replicates.

Supplementary Figure 7

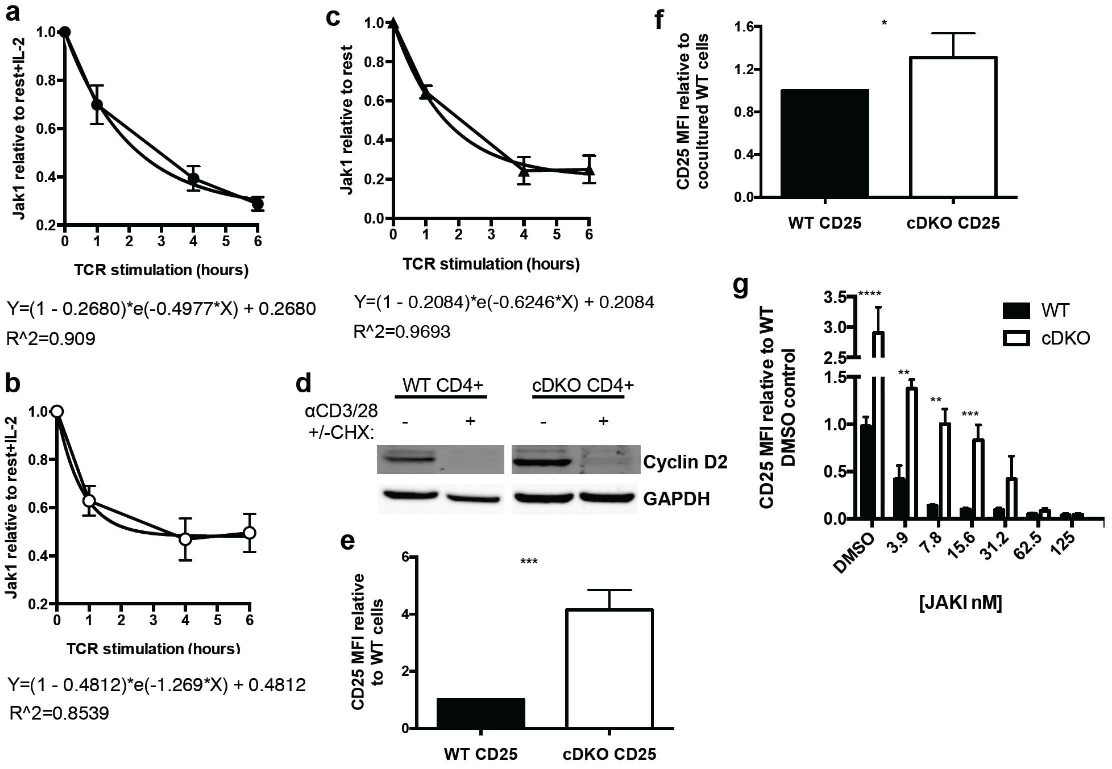

Supplementary Figure 7. Ndfip1/Ndfip2 deficient T cells have aborted Jak1 degradation and increased protein expression of STAT5 target genes.
(**a,b**) Jak1 degradation as shown in **Figure 7g** for WT (**a**) and cDKO (**b**) CD4+ T cells fits to significantly different one phase exponential decay curves (p=.0201). (**c**) Jak1 degradation in *Ndfip2*-/- cells is not significantly different from WT cells. (**d-g**) STAT5 targets show increased expression in DKO CD4+ T cells. (**d**) Cyclin D2 immunoblotting in restimulated DKO and WT CD4+ T cells. Cycloheximide was added 2hrs after αCD3/CD28 stimulation; cells were then incubated an additional 2hrs. Representative of 5 biologic replicates (either cDKO or *Ndfip1/Ndfip2*-/-). (**e-g**) Naïve CD4+ T cells were sorted from age-matched cDKO and control mice, CFSE labeled, and stimulated for 5 days with plate-bound αCD3/CD28. (**e**) CD25 was evaluated on day 5 by flow cytometry and quantified relative to the MFI of CD25 on experiment matched WT CD4+ T cells. (**f**) Naïve CD4+ T cells were sorted from age-matched cDKO and congenic CD45.1 WT mice, mixed in a 1:1 ratio, CFSE labeled, and stimulated for 5 days with plate-bound αCD3/CD28. CD25 was assessed as in (**e**). (**g**) CD25 MFI on WT and cDKO CD4+ T cells cultured as in (**e**) in the presence of increasing amounts of Jak inhibitor I, quantified relative to experiment matched untreated WT cell CD25 MFI. (**e,f**) Quantified relative to experiment matched WT cells, average shown +/-SEM, n=6-17. P values calculated by one sample t-test against the normalized WT value of 1. (**g**) Quantified relative to experiment matched untreated WT cells, average shown +/-SEM, n=3-6 at each inhibitor dose. P values calculated by unpaired two sample t-test. * $p<0.05$, ** $p<0.01$, ***$p<0.001$, ****$p<0.0001$.

Supplementary Figure 8

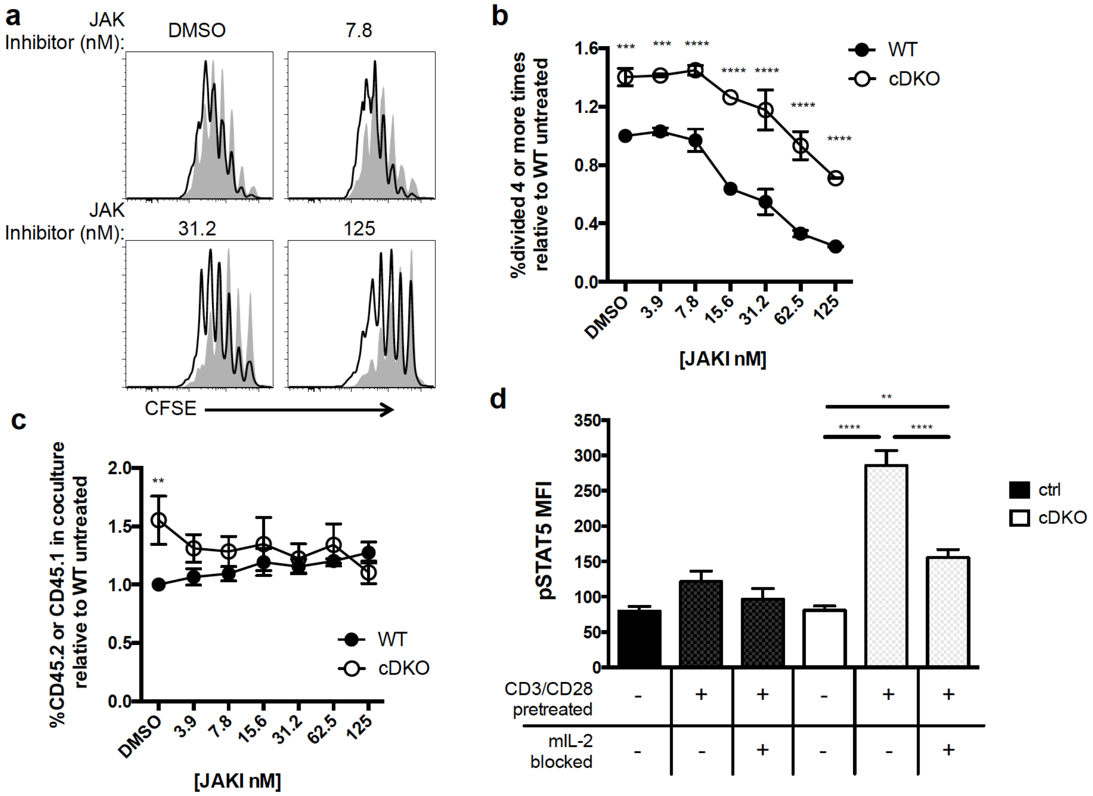

Supplementary Figure 8. Ndfip1/Ndfip2 deficient T cells respond to Jak inhibition in a cell-intrinsic fashion.
(**a-c**) Naïve CD4+ T cells were sorted from age-matched cDKO and congenic CD45.1 WT mice, mixed in a 1:1 ratio, CFSE labeled, and stimulated for 5 days with plate-bound αCD3/CD28 in the presence of increasing amounts of Jak inhibitor I. (**a**) Representative histograms of CFSE dilution in cocultured WT and cDKO CD4+ T cells in the absence or presence of a Jak inhibitor. Previously gated on live singlets, CD4+, CD45.2+ or CD45.1+ (**b,c**) Quantification of (**b**) cells divided 4+ times and (**c**) %CD45.2 or %CD45.1 on day 5 of coculture. (**b**) Quantification of cell division normalized to cocultured WT cells. (**b,c**) Quantifications shown as average +/- SEM from 4 biologic replicates. P values calculated by multiple t-test with Holm-Sidak correction. (**d**) Quantification of pSTAT5 staining in cDKO and WT CD4+ T cells rested in the absence of IL-2 overnight then treated +/- αCD3/CD28 beads in the presence or absence of αmouse IL-2 blocking antibody. Average MFI shown +/- SEM, n=5-7. P values calculated by two-way ANOVA. * $p<0.05$, ** $p<0.01$, ***$p<0.001$, ****$p<0.0001$.

Supplementary Figure 9

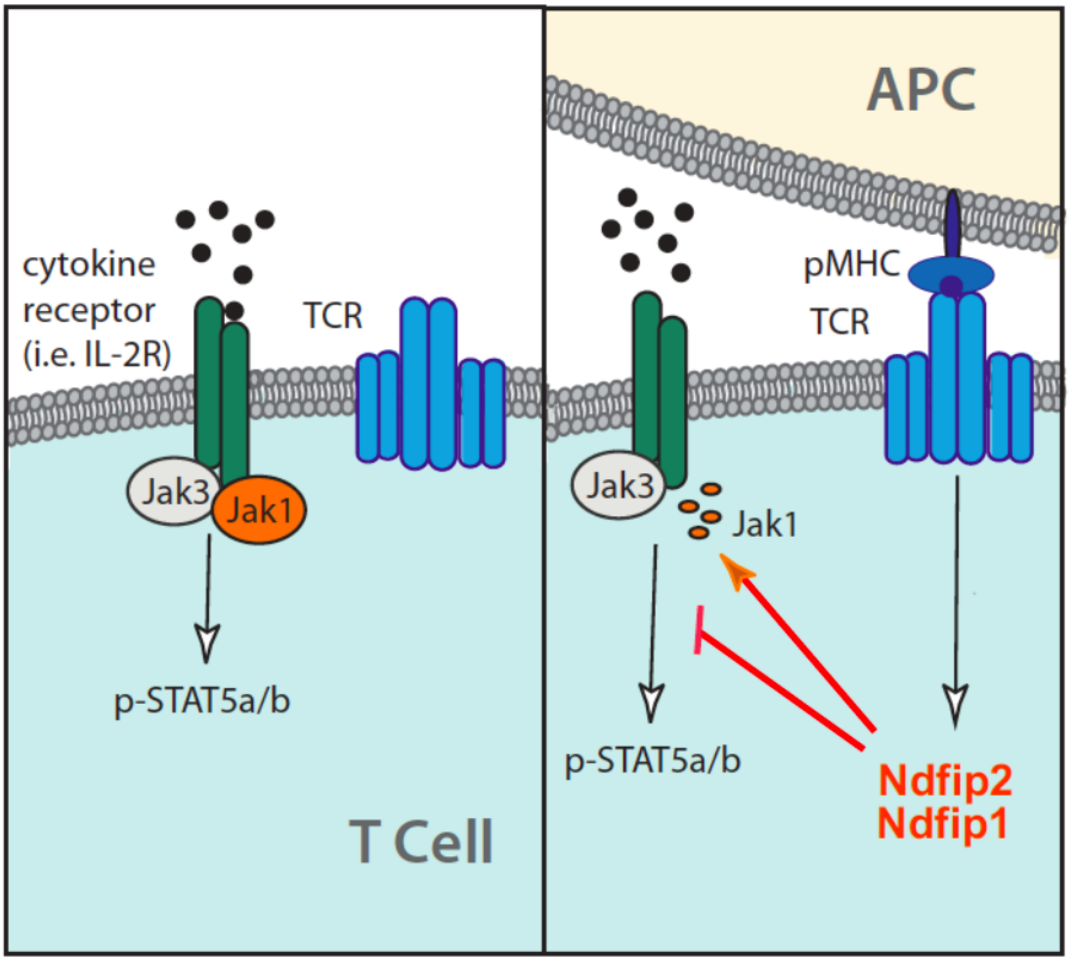

Supplementary Figure 9. Schematic illustrating how Ndfip1/Ndfip2 promote Jak degradation in restimulated CD4+ T cells.
In resting previously activated cells, cells receive survival and proliferation signals through common γ chain receptors (IL-2, IL-7, IL-15). In response to these Jak1/3 dependent cytokines, STAT proteins are phosphorylated to driver transcriptional programs and promote continued proliferation and survival. When the TCR is engaged, Jak1 is rapidly degraded in an Ndfip-dependent fashion, terminating cytokine signaling, even when the cytokine is still available. This prevents cytokine-driven survival and proliferation signals from “over-riding” signaling programs induced by the strength of TCR signal, thereby preventing aberrant activation/function of CD4+ T cells that fail to receive appropriate TCR signals.

Supplementary Figure 10

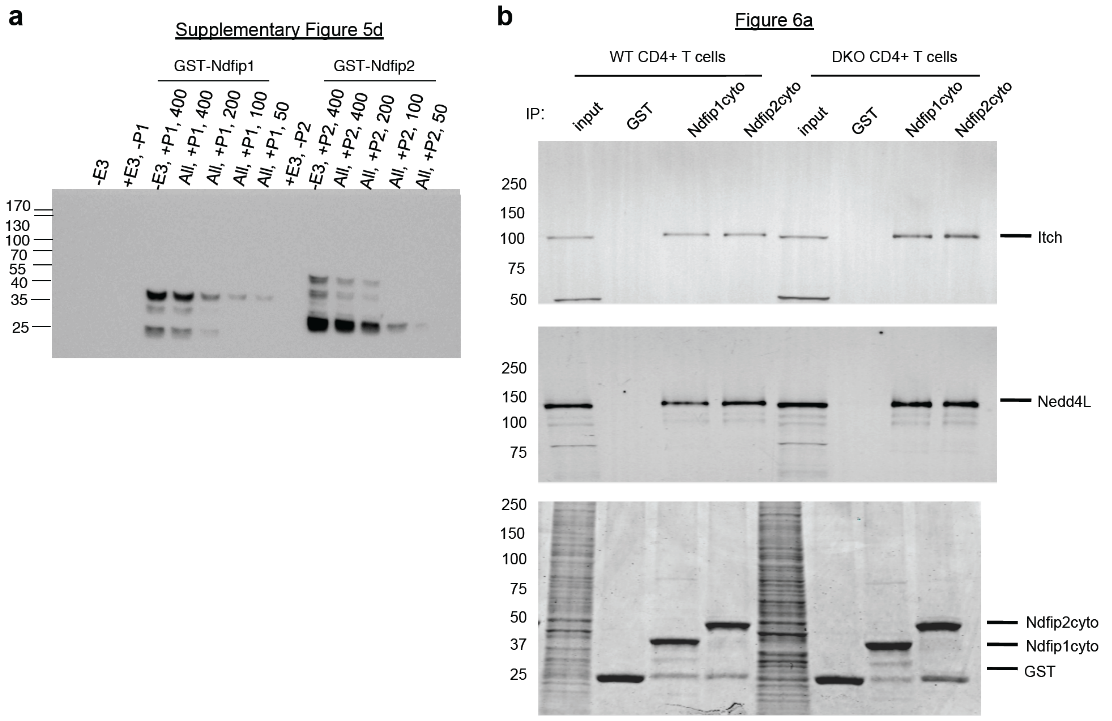

Supplementary Figure 11

a

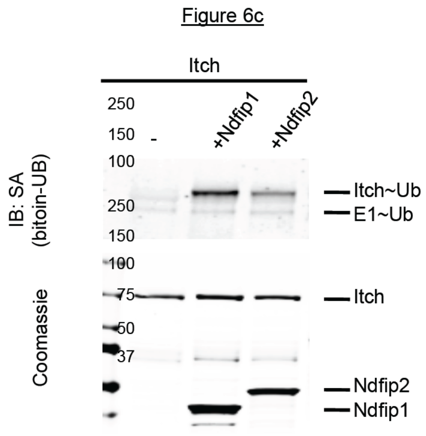

b

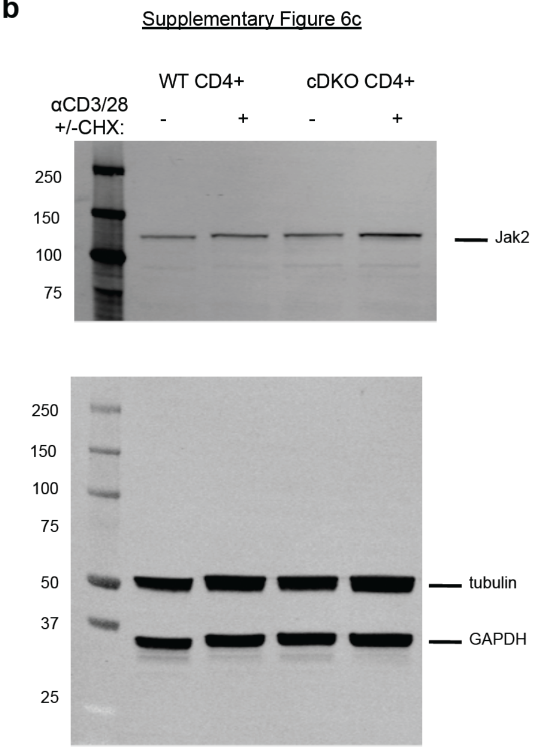

Supplementary Figure 12

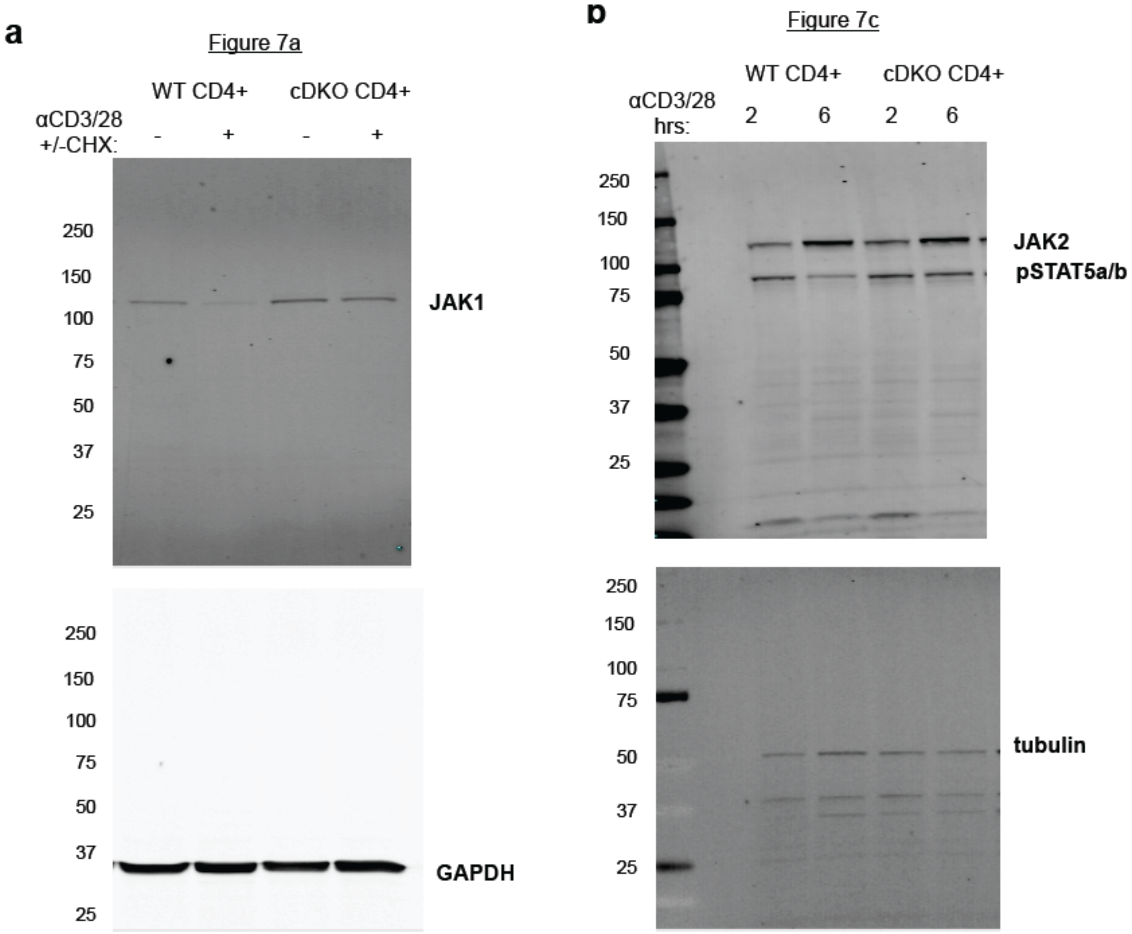

Supplementary Figure 13

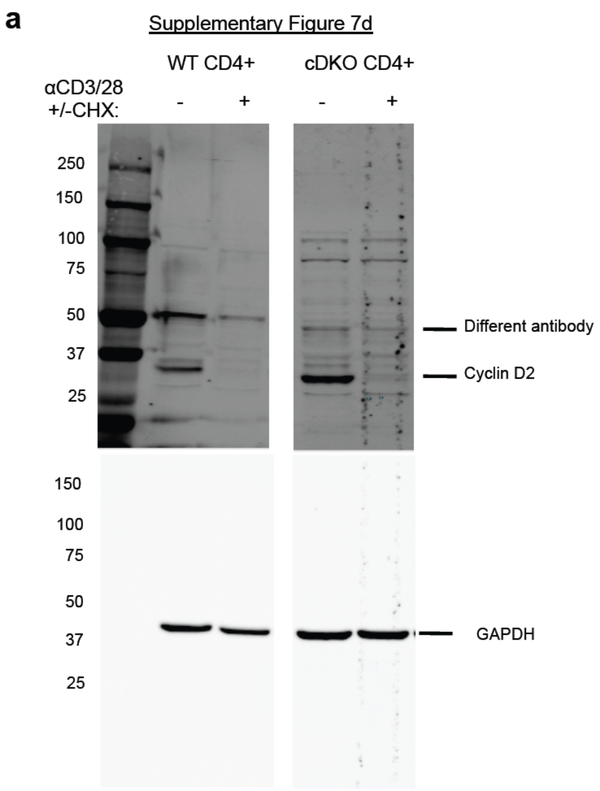

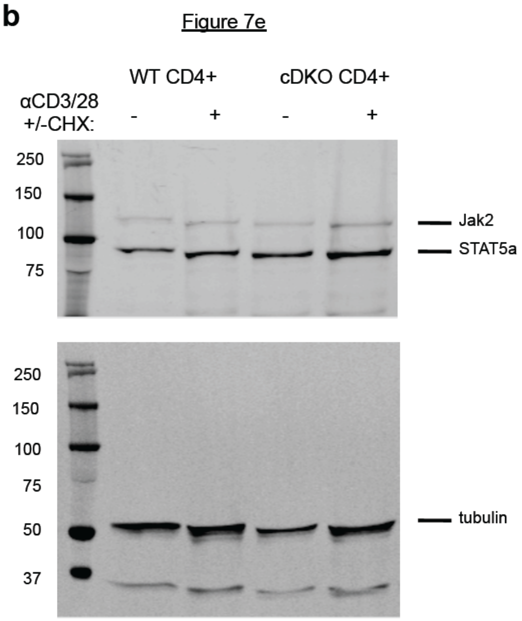

Supplementary Table 1. Fetal genotyping of intercrossed *Ndfip2-/-Ndfip1+/-*, *Ndfip2+/-Ndfip1+/-* mice

Ndfip1, Ndfip2[a,b]	Observed	Expected[c]
**-/- -/-**	**7.5%**	**12.5%**
+/- -/-	22.5%	25.0%
+/+ -/-	10.0%	12.5%
-/- +/-	12.5%	12.5%
+/- +/-	25.0%	25.0%
+/+ +/-	22.5%	12.5%

[a]Genotyping results from tail biopsy gDNA, embryonic day 18-20 fetuses
[b]40 pups genotypes
[c]Expected frequency based on Mendelian inheritance of *Ndfip1* and *Ndfip2* alleles
